# Patient‐Reported and Clinician‐Reported Esthetic Outcomes at Implant Sites Are Not Associated: A Systematic Review With Individual Participant Data Meta‐Analysis

**DOI:** 10.1111/clr.70019

**Published:** 2025-08-20

**Authors:** Sofya Sadilina, Nicolas P. A. Müller, Franz J. Strauss, Ronald E. Jung, Daniel S. Thoma, Stefan P. Bienz

**Affiliations:** ^1^ Clinic for Reconstructive Dentistry University of Zurich Zurich Switzerland; ^2^ Department of Periodontology, Research Institute for Periodontal Regeneration Yonsei University College of Dentistry Seoul Republic of Korea; ^3^ Universidad Autonoma de Chile Santiago Chile

**Keywords:** clinician‐reported outcomes, dental implants, dentistry, esthetic outcomes, patient‐reported outcomes, systematic review

## Abstract

**Objectives:**

This systematic review aimed to determine whether patient‐reported outcomes (PROs) are associated with clinician‐reported outcomes (ClinROs) in terms of esthetics in patients with single implant‐supported crowns in the esthetic region.

**Methods:**

A systematic electronic search was conducted following a pre‐established protocol to identify randomized controlled trials (RCT) involving patients with single implant‐supported crowns in the esthetic region. Studies had to assess both patient‐ and clinician‐reported outcomes. A two‐stage individual participant data (IPD) meta‐analysis was conducted. First, each study was analyzed separately to obtain correlation coefficients. Second, these estimates were pooled using a random‐effects restricted maximum likelihood (REML) model.

**Results:**

A total of 29 RCTs evaluating 1414 implant‐supported crowns were included, with IPD available for 14 trials evaluating 675 patients. At crown insertion, IPD meta‐analysis from 171 patients across four RCTs showed no significant correlations (*r* = 0.11, 95% CI [−0.04; 0.27], *p* = 0.16) between pink esthetic score (PES) and patient satisfaction with esthetics assessed with visual analogue scale (VAS). At the 1‐year follow‐up, IPD from 502 patients in 11 studies showed a negligible positive correlation (*r* = 0.09, 95% CI [−0.00; 0.18], *p* = 0.06) between PES or modified PES and VAS esthetic satisfaction. At 10‐year follow‐up, data from 80 patients in two studies showed no correlation between modified PES and VAS patient satisfaction (*r* = −0.05, 95% CI [−0.37; 0.27], *p* = 0.75). Regarding white esthetic score (WES) and VAS satisfaction, data from 376 patients in seven studies showed no significant correlations at the 1‐year follow‐up (*r* = 0.03, 95% CI [−0.08; 0.13], *p* = 0.60).

**Conclusion:**

Clinician‐reported outcomes, using PES and WES, showed no correlation with patient‐reported esthetic satisfaction, regardless of the follow‐up duration.

**Trial Registration:**

PROSPERO number CRD42023394920

## 1 Introduction

Within the last two decades, the value of esthetic outcomes significantly increased in medical and dental fields, and esthetic assessments became a criterion to rate treatment success (Cosyn et al. 2017; Pjetursson et al. 2014). For current treatments, the goal is to create implant‐supported restorations resembling natural teeth, including the surrounding soft tissues (Studer et al. 1996; Sadilina et al. 2024). Nevertheless, little is known about whether the clinicians' esthetic evaluations align with patient perceptions (Cosyn et al. 2017).

Esthetic assessments at implant sites can be categorized into metric parameters (e.g., mid‐facial recession measured in millimetres) and esthetic indexes (e.g., Pink Esthetic Score [PES]) (Cosyn et al. 2022). Among these indexes, PES is the most well‐known to assess esthetics around a dental implant. As a clinician‐reported outcome (ClinROs), PES has demonstrated favorable reliability and consistency across different studies, aiming to provide reproducible data (Hof et al. 2018). However, the association between PES and patients' perception remains unclear. Esthetic parameters are challenging to assess, and even validated esthetic indices have rarely achieved exact reproducibility. Moreover, subjective factors could be influenced by the patient's and clinician's expectations (Bienz et al. 2022). Previous studies have indicated a discrepancy between clinicians and patients, with the latter tending to be less critical of aesthetic outcomes (Thoma et al. 2024; Meijndert et al. 2007). It is presumed that patient expectations and psychological factors influence this discrepancy, yet robust evidence supporting this assumption is limited.

To address this, patient‐reported outcomes (PROs) are increasingly used in treatment selection and outcome assessment, especially in cases with high esthetic demands. PROs are measured by patient‐reported outcomes measures (PROMs) (Powers 3rd et al. 2017). PROMs are questionnaires to assess subjective patient's perception regarding various aspects of their health and the impact of a disease or its treatment on quality of life (McGrath et al. 2012). PROMs represent an average of what patients value, and they are becoming a decisive factor for treatment planning (Thoma and Strauss 2022). Given that esthetic expectations depend on the tooth position and visibility during smiling, PROMs are particularly valuable in the esthetic zone, where patient's subjective evaluation comes first.

However, the data from objective and subjective criteria may differ. Little investigation has been done so far to verify whether the objective parameters obtained by clinicians coincide with the perception of the patient. To summarize the variety of esthetic outcomes, including secondary outcomes, the present systematic review gathered data reporting PROMs and ClinROs from the same patient. Given the limitations of previous meta‐analyses using aggregated data in related fields, an individual participant data (IPD) meta‐analysis is warranted (Riley et al. 2010). IPD offers several advantages. First, pooling individual‐level data allows the extraction of esthetic outcomes that may have been reported only as secondary or additional outcomes, thereby increasing statistical power. Second, IPD reduces aggregation bias, and the larger number of participant‐level data points enables a more comprehensive assessment of individual‐level cofactors.

Therefore, the aim of the present systematic review and IPD meta‐analysis of randomized controlled trials was to assess the association of PROs and ClinROs on the esthetics of implant‐supported restorations in the esthetic region.

## 2 Material and Methods

### 2.1 Protocol and Registration

This manuscript was reported according to the Preferred Reporting Items for Systematic review and Meta‐Analysis of Individual Participants Data (PRISMA‐IPD) (Stewart et al. 2015) and the 2021 Cochrane collaboration guideline (Higgins et al. 2021). A detailed protocol was designed before the start of this review and prospectively registered on the International Prospective Register of Systematic Reviews (PROSPERO ID: CRD42023394920). Ethical approval was not required for this meta‐analysis; although each of the included studies was approved by the institutional ethics committee of the participating site.

### 2.2 Terminology

Because of the heterogeneity in the terminology reported in the existing literature, the following definitions were used according to the Cochrane Handbook for Systematic Reviews of Interventions (Johnston et al. 2019).

Patient‐reported outcome (PRO) is “any report of the status of a patient's health condition that comes directly from the patient without interpretation of the patient's response by a clinician or anyone else” (U.S. Food and Drug Administration, F 2021b).

Clinician‐reported outcomes (ClinROs) are “measurements based on a report that comes from a trained health‐care professional after observation of a patient's health condition” (U.S. Food and Drug Administration, F 2021a). Most ClinROs measures involve a clinical judgment or interpretation of the observable signs, behaviors, or other manifestations related to a disease or condition FDA‐NIH Biomarker Working Group 2016. BEST (Biomarkers, EndpointS, and other Tools). In this systematic review, ClinROs were evaluated with the scope of objective indexes and subjective factors related to the clinician's perception of esthetics.

### 2.3 Participants‐Intervention‐Outcome‐Study Design (PIOs)

The eligibility criteria of the current systematic review were organized by the modified PICO acronym, excluding the “comparison” element, and were aimed to answer the following focused question: “In patients with an implant‐supported crown in the esthetic region, are patient‐reported outcomes associated with clinician reported outcomes in terms of esthetic outcomes?”

- Population: Partially edentulous adult patients with at least one implant‐supported crown surrounded by neighboring teeth in the esthetic region.
- Intervention: An implant‐supported crown that has been rated in regard to satisfaction and esthetics by the patient as well as the clinician or professional examiner.
- Outcomes: Patient‐reported outcome, measured with Visual Analogue Scale (VAS), NRS (Numeric Rating Scale), OHIP (Oral Health Impact Profile) or other types of standardized questionnaires with more than a 3‐point scale; and clinician‐reported outcomes, measured with Pink Esthetic Score (PES), White Esthetic Score (WES), VAS, mid‐facial recession, color measurements, or other types of standardized esthetic indices.
- Study design: Only randomized controlled trials (RCT) either with parallel or split‐mouth design were evaluated to maintain consistency in quality assessment criteria and to reduce potential risk of bias. No studies were excluded on the basis of publication date, publication status (ahead to print) or number of included patients.

We assessed study eligibility criteria based on the PIO framework (Table 1). All decisions regarding the eligibility criteria were made by the group of review authors, including dentists with professional experience in prosthetic and implant dentistry.

**TABLE 1:** Summary of review search strategy and eligibility criteria.

Databases	Medline (via Pubmed) Embase Web of Science
Registers	Cochrane central register of controlled trials (CENTRAL)
Other sources	Forward and backward reference checking from reviewed papers and relevant systematic reviews on the topic
Key terms	**Population (P):** (dental implants) OR (dental implantation) OR (dental prothesis, implant‐supported) **AND** (patient reported outcome measures) OR (PROMS) OR (patient satisfaction) OR (patient expectation) OR (patient centered outcome) OR (patient opinion) OR (patient‐reported) OR (patient related) OR (visual analog scale) OR (VAS) OR (quality of life) OR (numerical rating scale) OR (OHIP) OR (Questionnaire) **AND** (clinician reported outcomes) OR (operator reported outcome measures) OR (esthetics, dental) OR (aesthet*) OR (pink esthetic score) OR (PES) OR (white esthetic score) OR (WES) OR (esthetic outcomes) OR (esthetic index) OR (visual analog scale) OR (VAS).
Limits	
Dates	Any publication date until search period
Language	English, German, Spanish, Russian
Location	International
Article type	Peer reviewed articles
Eligibility criteria	
Types of studies	Only RCTs. Methodological quality–not used as an exclusion criterion but considered when synthetizing the evidence for all studies.
Inclusion	Population: Systematically health patients, older than 18 years and with presence of at least one implant‐supported fixed dental restoration in the esthetic region (15–25); Intervention: An implant‐supported crown that has been rated in regards to satisfaction and esthetics by the patient as well as clinician or professional examiner; Outcome: PROMs, measured with VAS, NRS, OHIP or other types of standardized questionnaires with more than a 3‐point scale and ClinROs, measured with PES, WES, VAS, mid‐facial recession, color measurements or other type of standardized esthetic indices; Study design: only RCT, either with parallel or split‐mouth design.
Exclusion	(1) Unsuitable population (not evaluating single implant‐supported crown surrounded by neighbouring teeth in the maxillary esthetic region); (2) Not evaluating PROMs measured by more than a 3‐point scale or 3‐item standardized questionnaire; (3) Not evaluating esthetic outcomes; (4) Evaluating PROMs and ClinROs registered in different time points; (5) Inadequate study type (not RCT); (6) Articles reported not in English, German, Spanish or Russian; (7) Trial registered, not yet published; (8) Trial of published and included articles.
Search date	January 20, 2025

Abbreviations: ClinROs, clinician‐reported outcomes; NRS, numerical rating scale; OHIP, oral health impact profile; PES, pink esthetic score; PROMs, patient reported outcome measurements; RCT, randomized controlled trial; VAS, visual analogue scale; WES, white esthetic score.

### 2.4 Search Strategy

Two review authors (SS and FS) conducted in duplicate electronic searches in MEDLINE (searched via PubMed), The Cochrane Central Register of Controlled Trials, Embase, and Web of Science up to January 20, 2025. The search strategy was designed and adapted to each type of database (Table S1). No search filters were applied. In addition, cross‐reference checks were conducted within the bibliographies of all the included studies and within the relevant reviews on the topic (De Bruyn et al. 2015; Wittneben et al. 2018; Wittneben et al. 2023; Younes et al. 2018).

### 2.5 Study Selection

Based on the inclusion and exclusion criteria (Table 1), two reviewers (SS and NM) independently screened all titles and abstracts using the Rayyan Online Software (Qatar Computing Research Institute), while the first 100 abstracts were discussed for calibration purposes prior to formal selection. No restrictions were made in terms of study duration, but the language was restricted to English, German, Spanish, and Russian. Any disagreement between the two reviewers was discussed and resolved by a third review author (SB). Subsequently, the same authors conducted a full‐text analysis of studies identified as potentially eligible, after calibration of the first five articles. Any conflicts were resolved by a third review author (SB). The inter‐agreement between reviewers was quantified using Cohen's Kappa score.

### 2.6 Data Extraction

Corresponding authors of the included articles were contacted by e‐mails and were kindly asked to provide a table with the raw data for all the patients regarding utilized PROMs and ClinROs. A second e‐mail was sent after 2 weeks. For the included studies of which raw data was not available, means and standard deviations were extracted from the publication. For each eligible trial, two reviewers (SS and NM) extracted the data independently without modifications using a standardized, pilot‐tested data extraction table (Excel Microsoft Corporation) after calibration. In cases of multiple publications from trials evaluating the same cohort, the information was extracted from all the reports directly into a single data collection form and followed a two‐step approach. The general characteristics (e.g., study type, population characteristics) were obtained once from the primary study, and the specific outcomes (e.g., ClinROs assessment in different time points) were extracted from all relevant publications, including different follow‐up periods.

### 2.7 Assessment of Risk of Bias and Quality of Evidence

For each included study, two authors (SS and NM) independently used the risk of bias assessment for randomized controlled trials tool 2 (ROB‐2) from the current version of the Cochrane handbook (Sterne et al. 2019). In case of disagreement, both reviewers reached agreement on any assessment, resolved by the third author (FS).

The quality of evidence for each estimated effect size was evaluated using GRADE guidelines (Schunemann et al. 2008), considering factors such as inconsistencies in studies results, indirectness of evidence, and study imprecision. The findings were summarized in a GRADE evidence profile table. The GRADE guidelines were used to categorize heterogeneity, with < 40% indicating low, 30%–60% representing moderate, 50%–90% signifying substantial, and 75%–100% reflecting considerable heterogeneity (Guyatt et al. 2011).

### 2.8 Data Synthesis

The descriptive and qualitative aspects of the included studies were summarized, covering factors such as RCT type, population characteristics, type of augmentation, evaluated timepoints, and details on patient and clinician‐reported outcome measures. To ensure analytical consistency, patient‐reported outcome measures utilizing different measuring units were standardized prior to statistical analysis (e.g., 10 cm VAS scale were converted to 100 mm).

### 2.9 Data Analysis

For this meta‐analysis, we conducted a two‐stage individual participant data (IPD) meta‐analysis. In the first stage, each study was analyzed separately to obtain correlation coefficient estimates and their variances (Burke et al. 2017). Spearman correlation coefficients were calculated from the original raw data. In the second stage, we pooled the correlation estimates across studies using a random‐effects via Restricted Maximum Likelihood (REML) model to account for potential heterogeneity (Poeppl et al. 2007; Veroniki et al. 2016). To ensure valid inference, Fisher's *Z* transformation was applied before pooling, as it approximates a normal distribution, which is essential when combining correlation estimates (Rosner and Glynn 2007). This approach allowed us to compute 95% confidence intervals and pooled effect estimates while minimizing bias from non‐normality in correlation distributions.

If a study examined the correlation between PROMs and more than one scale (e.g., PES or modified PES) a subgroup analysis was performed.

### 2.10 Subgroup and Heterogeneity Analysis

Subgroup analyses were conducted to examine the impact of follow‐up time on correlations. We assessed between‐study heterogeneity by calculating the Q statistic, derived from the chi‐squared test, and the inconsistency index (*I* ^2^). A threshold of *I* ^2^ > 50% was set to indicate significant heterogeneity, with a *p*‐value < 0.10 considered statistically significant. The *τ* ^2^ value of 0.01 suggests a small amount of variance in effect sizes between studies (Higgins and Thompson 2002).

A correlation coefficient was considered between 0.0 and 0.09 to be negligible, 0.10 and 0.39 to be weak, 0.40 and 0.69 to be moderate, 0.70 and 0.89 to be strong, and 0.90 and 1.0 to be a very strong correlation (Schober et al. 2018). All analyses were conducted using STATA version 18.5 (StataCorp, College Station, TX).

## 3 Results

### 3.1 Search

**FIGURE 1:**
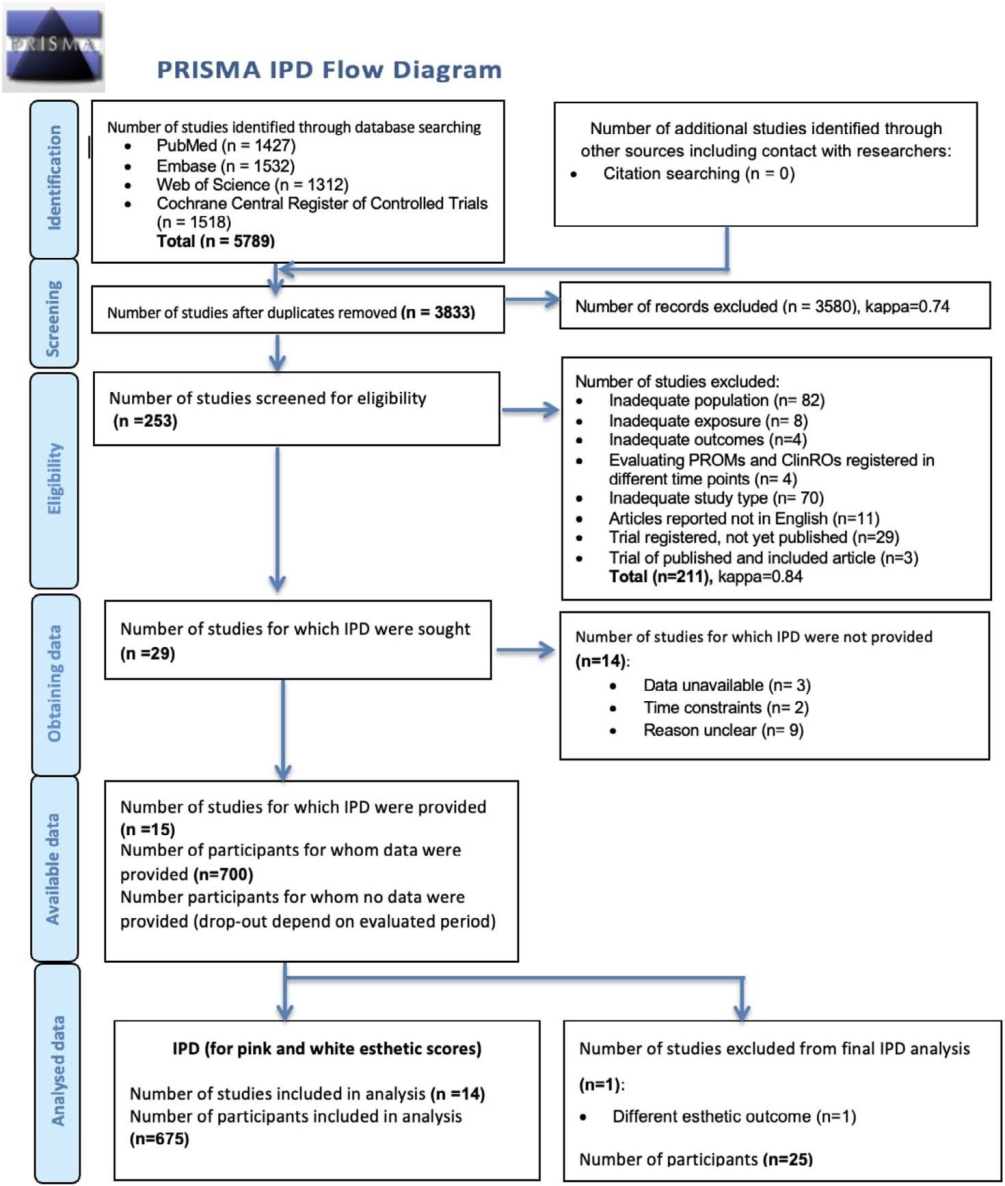
PRISMA‐IPD flow chart for the review. IPD, individual patient data.

The initial search yielded 5789 publications. of which 1427 were retrieved from Medline (via PubMed), 1532 entries from Embase, 1518 trials from CENTRAL, and 1312 studies via Web of Science. After excluding 1956 duplicates, the total number of entries was 3833. Of these, 3580 were discarded after reviewing the titles and abstracts. In total, 253 publications were selected for full‐text analysis, while 211 were excluded during this stage (the list of excluded studies and corresponding reasons were reported in Table S2). No additional studies were identified through cross‐reference checking. The inter‐rater agreement during the title and abstract screening phase was substantial agreement between reviewers (*k* = 0.74, agreement 84%), and it remained high during the full‐text analysis phase (*k* = 0.84, agreement 90%). Finally, 42 articles reporting on 29 unique patient populations were included (Figure 1).

**FIGURE 2:**
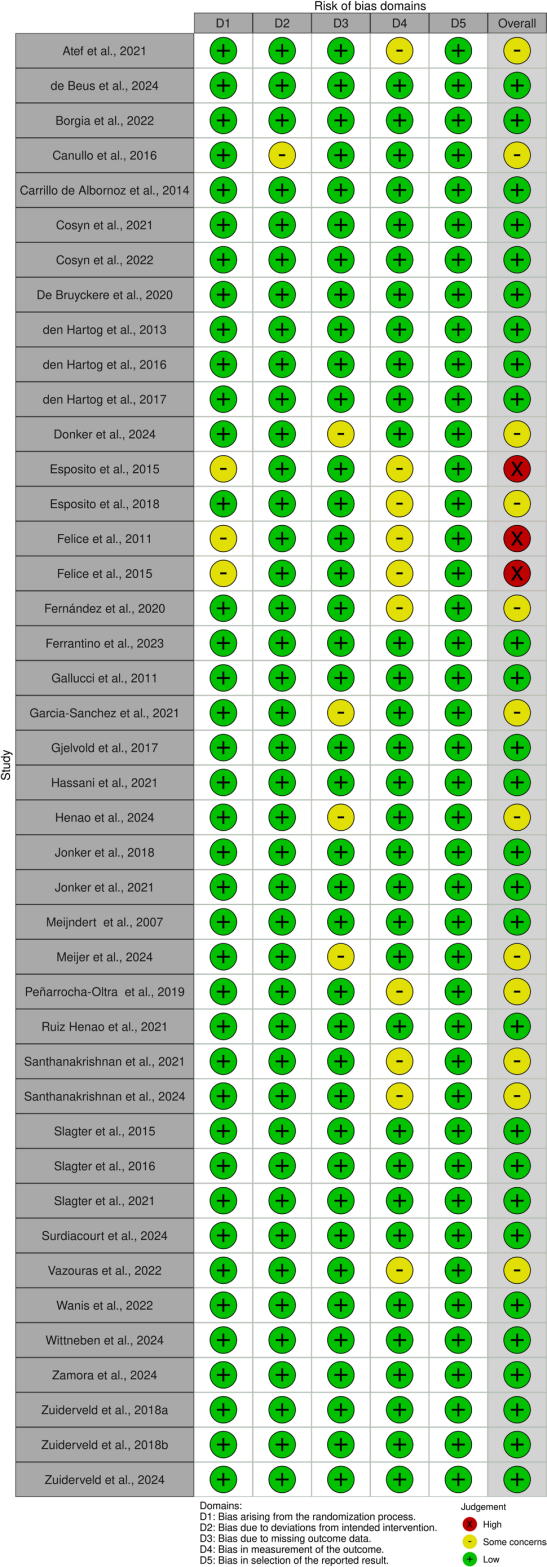
Risk of bias assessment for randomized controlled trials.

Table 2 summarizes the general details, participant information, and implant characteristics of the included studies. Overall, 1414 patients and 1414 implant‐supported crowns were part of the analysis. According to the summary of the quality assessment by the ROB‐2 tool, 27 articles showed a low risk of bias; 12 demonstrated some concerns; and three studies showed a high risk of bias (Figure 2). Ten RCTs revealed some concerns related to the bias in the measurement of the outcome (domain 4). Three studies had some concerns associated with the randomization process (domain 1) and one trial due to deviations from intended interventions (domain 2). Four RCTs demonstrated some concerns because of missing outcome data (domain 3); most of them were the long‐term follow‐ups of the original trial.

**TABLE 2:** Characteristics of the included studies–Participants and implants.

*N*	Study/Country	RCT type	Participants			Implants		
			Number of patients (implants)	Age, years, mean [SD] (range)	Gender (male/female)	Type of intervention	Type of augmentation	Implant brand
1	Atef et al. 2021/Egypt	Two arms, parallel	42 (42)	36 [5.5]	*10/30*	Intervention‐1: socket shield; Intervention‐2: GBR with xenograft	Intervention‐2: bone	ISII, Neobiotech
2	de Beus et al. 2024/Netherlands	Two arms, parallel	49 (49)	Intervention‐1: 52.4 (23.1–72.5) Intervention‐2: 52.1 (28.1–76.3)	24/25	Intervention‐1: titanium impl Intervention‐2: zirconia impl	No	One arm: Straumann Pure, Straumann; Second arm: SLActive, Straumann BL RC, Straumann
3	Borgia et al. 2022/Uruguay	Two arms, parallel	31 (31)	Intervention‐1: 51.8 [10.9] Intervention‐2: 55.6 [11.8]	10/21	Intervention‐1: GBR with autologous bone from tuberosity; Intervention‐2: GBR with DBBM	Bone	Osseotite Certain prevail, Biomet 3i
4	Canullo et al. 2016/Spain	Two arms, parallel	30 (30)	58.2 (31–79)	18/12	Intervention −1: cleaning protocol by steaming Intervention‐2: plasma of argon treatment	No	Premium SP Implants, Sweden & Martina
5	Carrillo de Albornoz et al. 2014; Ferrantino et al. 2023/ Spain	Two arms, parallel	25 (25)	Intervention‐1: 51.8 [10.9] Intervention‐2: 51.6 [8.9]	11/15	Intervention‐1: titanium abutment Intervention‐2: zirconia abutment	No	Element RC; Thommen Medical AG
6	Cosyn et al., 2021; Cosyn et al. 2022; Surdiacourt et al. 2024/Belgium	Two arms, parallel	60 (60)	Intervention‐1: 50.1 [17.0] Intervention‐2: 48.2 [16.3]	29/31	Intervention‐1: soft tissue augmentation with CTG Intervention‐2: soft tissue augmentation with CMX	Soft tissue	Nobel Replace, Nobel Biocare
7	De Bruyckere et al. 2020/Belgium	Two arms, parallel	40 (40)	Intervention‐1: 51.0 [13.0] Intervention‐2: 48.0 [15.0]	21/19	Intervention‐1: Imp placement and GBR Intervention‐2: Impl placement and CTG	Intervention‐2: soft tissue; Intervention‐1: bone	NobelActive, Nobel Biocare
8	den Hartog et al. 2013; den Hartog et al. 2017/Netherlands	Three arms, parallel	92 (92)	Intervention‐1: 7.2 [12.9]; Intervention‐2: 40.1 [14.4]; Intervention‐3: 40.1 [17.2]	46/47	Intervention‐1: “smooth” implant neck Intervention‐2: “rough” implant neck Intervention‐3: “scalloped” implant neck	Deferent augmetation types within one study	Nobel Biocare, Nobel Biocare
9	den Hartog et al. 2016/Netherlands	Two arms, parallel	62 (62)	Intervention‐1: 40.1 [14.4] Intervention‐2: 38.4 [14.0]	26/36	Intervention‐1: conventional impl loading after 3 months Intervention‐2: immediate impl loading with temproray crown withing 24 h	Bone (if needed)	Nobel Replace Tapered Groove implant, Nobel Biocare
10	Esposito et al. 2018; Peñarrocha‐Oltra et al. 2019; Fernández et al. 2020/Spain	Two arms, parallel	30 (30)	Intervention‐1: 47.5 (23–70) Intervention‐2: 47 (20–65)	11/19	Intervention‐1: impl positioned in the natural “centra” position Intervention‐2: impl positioned about 3 mm more palatally	Bone (if needed)	Ticare Inhex implants, Mozo‐Grau Ticare
11	Felice et al. 2015/Italy	Two arms, parallel	50 (50)	Intervention‐1: 53.0 (39–72) Intervention‐2: 51.3 (32–71)	25/25	Intervention‐1: ARP with algae‐derived (phycogenic) bone substitute; Intervention‐2: immediate impl placement	Intervention‐1: ARP	XiVE S plus, Dentsply Friadent
12	Felice et al. 2011; Esposito et al. 2015/Italy	Two arms, parallel	106 (106)	Intervention‐1: 50 (30–72) Intervention‐2: 48 (28–70)	46/60	Intervention‐1: ARP with DBBM; Intervention‐2: immediate implant placement	Intervention‐1: ARP	MegaGen implant, Gyeonbuk
13	Gallucci et al. 2011/Switzerland	Two arms, parallel	20 (20)	NR	NR	Intervention‐1: porcelain‐fused‐to‐ceramic single‐impl crown; Intervention‐2: all‐ceramic single‐impl crown	No	Standard plus, straumann
14	Garcia‐Sanchez et al. 2021/Spain	Two arms, parallel	28 (28)	Intervention‐1: 47 [10.2] Intervention‐2: 47 [10.8]	10/18	Intervention‐1: flap; Intervention‐2: minimal split‐ thickness envelope flap	No	Biomimetic OCEAN, Avinent implants
15	Gjelvold et al. 2017/Sweden	Two arms, parallel	50 (50)	Intervention‐1: 40.9 [15.5] Intervention‐2: 40.8 [13.3]	20/30	Intervention‐1: delayed loading after 4 months; Intervention‐2: immediate impl. loading with temporary crown	Bone (if needed)	Tapered Internal implants, Bio‐Horizons
16	Hassani et al. 2021/Iran	Two arms, parallel	40 (40)	Intervention‐1: 42.8 [3.4] Intervention‐2: 38.1 [4.9]	16/24	Intervention‐1: delayed loading after 3 months; Intervention‐2: immediate impl. loading with temporary crown within 48 h	Bone	Superline, dentium
17	Jonker et al. 2021/Netherlands	Three arms, parallel	75 (75)	Intervention‐1: 44 [12] Intervention‐2‐1: 50 [13] Intervention‐3: 49 [16]	33/42	Intervention‐1: spontaneous healing; Intervention‐2: ARP with DBBM covered with a palatal graft; Intervention‐3: ARP with DBBM covered by collagen matrix	Intervention‐1: No augmentation; Intervention‐2‐1ARP and soft tissue; Intervention‐3: ARP	Bone level tapered, SLActive, Straumann
18	Jonker et al. 2018/Netherlands	Two arms, parallel	52 (52)	Intervention‐1: 49.0 [14.1]; Intervention‐2: 45.0 [10.8]	30/22	Intervention‐1: ridge augmentation without resorbable membrane; Intervention‐2: ridge augmentation with resorbable membrane	Bone	Bone level, straumann
19	Meijndert et al. 2007/Netherlands	Three arms, parallel	93 (93)	33.3 (18–63)	44/49	Intervention‐1: GBR with autologous bone from chin; Intervention‐2: GBR with autologous bone from chin & DBBM; Intervention‐3: GBR with DBBM	Bone	Straumann
20	Ruiz Henao et al. 2021; Henao et al. 2024/Spain	Two arms, parallel	30 (30)	Intervention‐1: 56.0 Intervention‐2: 54.1	14/16	Intervention‐1: titanium impl; Intervention‐2: ceramic impl	Bone (if needed)	Intervention‐1: tissue level SLA titanium implants, Straumann; Intervention‐2: Straumann PURE Ceramic implants, Straumann
21	Santhanakrishnan et al. 2021; Santhanakrishnan et al. 2024/India	Three arms, parallel	75 (75)	Intervention‐1: 30.8 [6.5]; Intervention‐2‐1: 30.6 [6.3] Intervention‐3: 29.8 [9.7]	34/41	Intervention‐1: delayed impl placement within 4 months; Intervention‐2: socket shield; Intervention‐3: immediate impl. placement	Intervention‐2: bone	DIO implant system, Busan
22	Slagter et al. 2015; Slagter et al. 2021; Donker et al. 2024/Netherlands	Two arms, parallel	40 (40)	Intervention‐1: 42.3 [14.2] Intervention‐2: 39.5 [16.9]	13/27	Intervention‐1: delayed impl loading after 3 months; Intervention‐2: immediate impl loading with temporary crown	No	NobelActive TiUnite, Nobel Biocare
23	Slagter et al. 2016; Slagter et al. 2021; Meijer et al. 2024/Netherlands	Two arms, parallel	40 (40)	Intervention‐1: 48.6 [16.4] Intervention‐2: 43.7 [13.9]	18/22	Intervention‐1 delayed impl placement after ARP; Intervention‐2 immediate impl placement	Intervention‐1s: ARP	NobelActive, Nobel Biocare
24	Vazouras et al. 2022/USA	Three arms, cross‐over	25 (25)	43 (21–64)	8/17	Intervention‐1: gray titanium abutment; Intervention‐2 pink anodized titanium abutment; Intervention‐3 hybrid zirconia custimized abutment	No	Genesis, keystone dental
25	Wanis et al. 2022/Egypt	Two arms, parallel	24 (24)	Intervention‐1: 30.3 [6.6] Intervention‐2: 34.2 [7.9]	7/17	Intervention‐1: immediate impl placement with GBR and immediate loading with temporary crown; Intervention‐2: immediate impl placement with dual‐zone therapeutic concept	Intervention‐1: Bone	IS‐II implant, Neobiotech
26	Wittneben et al. 2023/Switzerland	Two arms, parallel	40 (40)	NR	NR	Intervention‐1 individualized CAD/CAM abutment; Intervention‐2 prefabricated stock abutment	No	Bone level implant; Straumann
27	Zamora et al. 2024 /Spain^a^	Three arms, parallel	45 (45)	Intervention‐1: 45.5 [13.9]; Intervention‐2‐1: 48.1 [13.8] Intervention‐3: 47.4 [16.2]	19/26	Intervention‐1: metal‐porcelain restoration; Intervention‐2: lithium disilicate restoration; Intervention‐3: porcelain‐layered, zirconium restoration	Soft tissue	Biomimetic OCEAN, Avinent
28	Zuiderveld et al. 2018a/Netherlands	Three arms, parallel	60 (60)	Intervention‐1: 42.0 [15.7] Intervention‐2:38.2 [16.7] Intervention‐3: 45.4 [17.0]	25/35	Intervention‐1: spontaneous healing; Intervention‐2: soft tissue augmentation with CTG from palate; Intervention‐3: soft tissue augmentation with XCM	Soft tissue	Nobel Replace CC, Nobel Biocare
29	Zuiderveld et al. 2018b; Zuiderveld et al. 2024/Netherlands	Two arms, parallel	60 (60)	Intervention‐2: 47.8 [16.5] Intervention‐2: 45.5 [15.5]	28/32	Intervention‐1: spontaneous healing; Intervention‐2: soft tissue augmentation with CTG from tuberosity	Intervention‐2: soft tissue	NobelActive, Nobel Biocare

Abbreviations: ARP, alveolar ridge preservation; CMX, collagen matrix; CTG, connective tissue graft; DBBM, deproteinized bovine bone mineral; GBR, guided bone regeneration; imp, implant; NR, not reported.

^a^ Data reported as a median.

### 3.2 Characteristics of the Included Studies

The included articles were published between 2007 and 2024. All RCTs had parallel design, and 22 studies included two arms, and seven trials investigated three arms. Ten articles were a follow‐up of previously published trials, with the investigated period from 1 to 5 years.

From the total of 1414 treated patients, 776 (54.9%) were females, 578 (40.9%) were males, and the gender of the remaining population (60 patients [4.2%]) was not reported. The included studies evaluated a variety of interventions. Among these, nine trials focused on implant loading protocols; four studies on abutment characteristics; four on bone augmentation; three on soft tissue augmentation; two on implant materials; and two on implant‐supported restoration material. Additionally, one study each investigated implant placement protocol, flap design, alveolar ridge preservation, implant position, cleaning protocol, and neck characteristics. Various types of bone and soft tissue augmentation within different time points were performed within included studies according to the proposed study design.

### 3.3 Patient‐Reported Outcomes (PROs) Regarding Esthetics

The identified PROs regarding esthetics in the selected literature were assessed with the following measuring tools:

1 PRO: Overall/general satisfaction/appearance/smile (*n* = 22)
  - Measuring instrument: Visual Analog Scale (VAS) (16/22); Unstandardized questionnaire (6/22).
2 PRO: Pink/gum/mucosal esthetics (*n* = 16)
  - Measuring instrument: Unstandardized questionnaire (7/16); VAS (9/16).
  2.1 PRO: Colour of gum (*n* = 7)
  - Measuring instrument: Unstandardized questionnaire (4/7); VAS (3/7).
  2.2 PRO: Shape of gum (*n* = 7)
  - Measuring instrument: Unstandardized questionnaire (4/7); VAS (3/7).
3 PRO: White/crown esthetics (*n* = 12)
  - Measuring instrument: Unstandardized questionnaire (4/12); VAS (8/12).
  3.1 PRO: Crown color (*n* = 7)
  - Measuring instrument: Unstandardized questionnaire (4/7); VAS (3/7).
  3.2 PRO: Crown shape (*n* = 7)
  - Measuring instrument: Unstandardized questionnaire (4/7); VAS (3/7).
4 PRO: esthetic satisfaction/appearance (*n* = 2)
  - Measuring instrument: Visual Analog Scale (VAS) (2/9).
5 PRO: Oral health‐related quality‐of‐life (*n* = 6)
  - Measuring instrument: OHIP‐14 (6/6).

Table 3 provides detailed qualitative information on the PROs and measuring instruments used in the selected studies. Moreover, one study evaluated the domain of overall satisfaction by several measuring tools.

**TABLE 3:** Characteristics of the included studies–Patient‐reported outcome measures and clinician‐reported outcome measures.

*N*	Study/Country	Characteristics					Results
		Patient‐reported outcome measures			Clinician reported outcome measures		
		PROMs scale (range, min/max)	Evaluated question, regarding esthetisc (key aspects)	Results, mean [SD] (range)	Results, mean [SD] (range)	Method of evaluation	Evaluated timepoint
1	Atef et al. 2021/Egypt	VAS (0/10 cm)	White esthetics Pink esthetics	Intervention‐1: 9.3 [0.9] Intervention‐2: 9.2 [0.7] Intervention‐1: 9.3 [0.1] Intervention‐2: 9.2 [0.7]	Midfacial recession: Intervention‐1: 0.4 [0.7] mm Intervention‐2: −0.4 [0.5] mm PES: Intervention‐1: 12.1 [0.6] Intervention‐2: 11.8 [0.3]	IOS and digital superimposition (Exocad, CAD software, Germany) Intra‐oral photographs	1Y FU
2	de Beus et al. 2024/Netherlands	Questionnarie Guljé et al. (0%–100%)	Crown color Crown shape Gum color Gum shape Overall satisfaction	NR NR NR NR Intervention‐1: 96.0 [5.3] Intervention‐2: 93.1 [4.3]	PES: Intervention‐1: 6.5 [1.6] Intervention‐2: 11.8 [0.3] PES/WES: Intervention‐1: 12.7 [4.2] Intervention‐2: 13.1 [3.0] WES: Intervention‐1: 6.2 [3.2] Intervention‐2: 6.6 [2.2]	Intra‐oral photographs Intra‐oral photographs Intra‐oral photographs	1Y FU
3	Borgia et al. 2022/Uruguay	VAS (0/10 cm) OHIP‐14 (0/56)	Overall satisfaction Health‐related quality	Unclear reported data	PES: Intervention‐1: 10.8 [1.9] Intervention‐2: 11.5 [1.7]	NR	1Y FU
4	Canullo et al. 2016/Spain	VAS (0/10 cm)	Overall satisfaction	Intervention‐1: 9.5 [2.8] Intervention‐2: 9.6 [4.1]	Buccal peri‐implant mucosa changes at the zenith: Intervention‐1: 0.2 [0.3] Intervention‐2: 0.5 [0.6]	Customized prefabricated stent	5Y FU
5	Carrillo de Albornoz et al. 2014; Ferrantino et al. 2023/Spain	Unstandarized question (1/6) VAS (0/10 cm)	Overall satisfaction Esthetics satisfaction	Unclear reported data Intervention‐1: 8.5 [1.6] Intervention‐2: 8.5 [1.7] Intervention‐1: 9.0 [1.4] Intervention‐2: 9.0 [1.7] Unclear reported data Intervention‐1: 9.0 [1.8] Intervention‐2: 8.8 [1.1]	ICAI‐crown: Intervention‐1: 3.4 [3.4] Intervention‐2: 2.0 [2.0] ICAI‐mucosa: Intervention‐1: 7.2 [2.7] Intervention‐2: 5.7 [3.0] ICAI‐total: Intervention‐1: 10.6 [4.4] Intervention‐2: 7.8 [3.2] ICAI‐crown: Intervention‐1: 4.4 [4.7] Intervention‐2: 2.2 [1.9] ICAI‐mucosa: Intervention‐1: 6.8 [3.1] Intervention‐2: 5.3 [3.0] ICAI‐total: Intervention‐1: 11.2 [5.4] Intervention‐2: 7.6 4 [3.5] ICAI‐crown: Intervention‐1: 5.6 [4.3] Intervention‐2: 4.1 [3.4] ICAI‐mucosa: Intervention‐1: 5.9 [2.2] Intervention‐2: 3.6 [1.3] ICAI‐total: Intervention‐1: 11.5 [5.6] Intervention‐2: 7.8 [3.5]	Intra‐oral photographs Intra‐oral photographs Intra‐oral photographs	1M FU 1Y FU 5Y FU
6	Cosyn et al., 2021; Cosyn et al. 2022; Surdiacourt et al. 2024/Belgium	VAS (0/100 mm)	Gums esthetics	Intervention‐1: 80.7 (71.8–89.5) Intervention‐2: 82.0 (73.3–90.7) Intervention‐1: 89.6 (82.3–96.9) Intervention‐2: 89.3 (82.0–96.6) Intervention‐1: 89.6 (82.8–96.3) Intervention‐2: 91.8 (85.0–98.5)	Midfacial recession: Intervention‐1: 0.1 mm (−0.4 to 0.7); Intervention‐2: 0.9 mm (0.3–1.5) Mucosal scarring index: Intervention‐1: 2.0 (1.6–2.4); Intervention‐2: 1.7 (1.3–2.1) PES: Intervention‐1: 10.4 (9.7–11.2) Intervention‐2: 10.5 (9.8–11.3) Midfacial recession: Intervention‐1: 0.3 (0.2–0.5); Intervention‐2: 0.3 (0.2–0.5) Mucosal Scarring Index: Intervention‐1: 1.3 (0.4–2.2); Intervention‐2: 1.3 (0.4–2.3) PES: Intervention‐1: 11.9 (10.8–13.1); Intervention‐2: 11.2 (10.1–12.3) Midfacial recession: Intervention‐1: 0.2 (0.0–0.5); Intervention‐2: 0.1 (0.0–0.4) Mucosal Scarring Index: Intervention‐1: 2.1 (1.4–2.7); Intervention‐2: 1.7 (1.0–2.4) PES: Intervention‐1: 10.9 (10.2–11.5); Intervention‐2: 10.5 (9.8–11.2)	IOS and digital superimposition (SMOP, Swissmeda, Switzerland) Intra‐oral photographs Intra‐oral photographs	3M FU 1Y FU 3Y FU
7	De Bruyckere et al. 2020/Belgium	VAS (0/100 mm) OHIP‐14 (0/56)	Soft tissue Crown Health‐related quality	Intervention‐1: 84 [14] Intervention‐2: 87 [15] Intervention‐1: 88 [10] Intervention‐2: 92 [5] Intervention‐1: 18.6 [5.5] Intervention‐2: 15.7 [2.3]	Mucosal scarring index: Intervention‐1: 2.5 [2.1]; Intervention‐2: 1.1 [1.3] PES: Intervention‐1: 10.1 [1.8]; Intervention‐2: 10.4 [2.2] WES: Intervention‐1: 8.7 [1.5]; Intervention‐2: 8.4 [1.1]	Intra‐oral photographs Intra‐oral photographs Intra‐oral photographs	1Y FU
8	den Hartog et al. 2013; den Hartog et al. 2017/Netherlands	Unstandarized questions (1/5) VAS (0/10 cm)	Color of the crown Shape of the crown Color of the gums Shape of the gums General satisfaction	Intervention‐1: 28 pat.‐5; Intervention‐2: 30 pat = 5; Intervention‐3: 28 pat = 5; Intervention‐1: 28 pat = 5; Intervention‐2: 31 pat = 5; Intervention‐3: 29 pat = 5; Intervention‐1: 26 pat = 5; Intervention‐2: 27 pat = 5; Intervention‐3: 26 pat = 5; Intervention‐1: 24 pat = 5; Intervention‐2: 27 pat = 5; Intervention‐3: 24 pat = 5; Intervention‐1: 8.8 [1.1]; Intervention‐2: 8.9 [1.0]; Intervention‐3: 9.1 [0.8] Intervention‐1: 8.4 [0.9]; Intervention‐2: 9.1 [0.9]; Intervention‐3: 8.6 [1.5]	ICAI crown: Intervention‐1: Excellent: 1 pat; Satisfactory: 17 pat; Moderate: 10 pat; Poor: 2 pat; Intervention‐2: Excellent: 1 pat; Satisfactory: 18 pat; Moderate: 10 pat; Poor: 2 pat; Intervention‐3: Excellent: 1 pat; Satisfactory: 19 pat; Moderate: 7 pat; Poor: 4 pat ICAI mucosa: Intervention‐1: Excellent: 2 pat; Satisfactory: 14 pat; Moderate: 8 pat; Poor: 6 pat; Intervention‐2: Satisfactory: 15 pat; Moderate: 6 pat; Poor: 10 pat; Intervention‐3: Satisfactory: 21 pat; Moderate: 4 pat; Poor: 6 pat; PES: Intervention‐1: 6.0 [1.9]; Intervention‐2: 6.3 [1.7]; Intervention‐3: 6.6 [1.6]; WES: Intervention‐1: 7.2 [1.5]; Intervention‐2: 7.4 [1.6]; Intervention‐3: 7.2 [1.6]; PES: Intervention‐1: 6.5 [1.3]; Intervention‐2: 6.8 [1.3]; Intervention‐3: 6.4 [1.5]; WES: Intervention‐1: 7.1 [1.4]; Intervention‐2: 7.7 [1.2]; Intervention‐3: 7.1 [1.4]	Intra‐oral photographs Intra‐oral photographs Intra‐oral photographs Intra‐oral photographs	1Y FU 5Y FU
9	den Hartog et al. 2016/Netherlands	Unstandarized question (1/5) VAS (0/100 mm)	Color of the crown Form of the crown Color of the mucosa around the crown Form of the mucosa around the crown Overall satisfaction	Intervention‐1: 100 Intervention‐2: 100 Intervention‐1: 96.7 Intervention‐2: 93.3 Intervention‐1: 90.3 Intervention‐2: 96.7 Intervention‐1: 86.6 Intervention‐2: 80.6 Intervention‐1: 89.5 [9.5] Intervention‐2: 91.5 [8.4] Intervention‐1: 96.8 Intervention‐2: 93.3 Intervention‐1: 100 Intervention‐2: 100 Intervention‐1: 87.0 Intervention‐2: 96.6 Intervention‐1: 87.0 Intervention‐2: 86.6 Intervention‐1: 89.0 [9.8] Intervention‐2: 92.7 [9.0] Intervention‐1: 96.4 Intervention‐2: 92.6 Intervention‐1: 89.3 Intervention‐2: 96.3 Intervention‐1: 92.9 Intervention‐2: 96.3 Intervention‐1: 82.1 Intervention‐2: 88.9 Intervention‐1: 91.3 [9.4] Intervention‐2: 93.5 [7.1]	Papilla index: Intervention‐1: 0–3.2; 1–27.5; 2–46.8; 4–22.6 Intervention‐2: 0–1.7; 1–28.3; 2–51.7; 4–18.3 Papilla index: Intervention‐1: 0–1.6; 1–19.4; 2–45.2; 4–33.9 Intervention‐2: 1–21.7; 2–35.0; 4–43.3 PES: Intervention‐1: 6.5 [1.6]; Intervention‐2: 7.1 [1.5]; WES: Intervention‐1: 7.6 [1.6]; Intervention‐2: 7.8 [1.5]; Papilla index: Intervention‐1: 1–22.2; 2–51.9; 4–25.9; Intervention‐2: 1–15.4; 2–48.0; 4–36.5 PES: Intervention‐1: 6.8 [1.3]; Intervention‐2: 7.2 [1.5]; WES: Intervention‐1: 7.7 [1.2]; Intervention‐2: 7.9 [1.2]	Clinically Intra‐oral photographs Intra‐oral photographs	CI 1Y FU 5Y FU
10	Esposito et al. 2018; Peñarrocha‐Oltra et al. 2019; Fernández et al. 2020/Spain	Unstandarized question (1/5)	Gum esthetics	Intervention‐1: 9 pat = 5; 1 pat = 4; Intervention‐2: 4 pat‐ 5, 3 pat‐ 4 Intervention‐1: 10 pat = 5; Intervention‐2: 9 pat‐5, 1 pat‐ 4 Intervention‐1: 10 pat = 5; Intervention‐2: 9 pat‐5, 1 pat‐ 4	PES: Intervention‐1: 10.0 [4.5] Intervention‐2: 6.0 [9.0] PES: Intervention‐1: 12.5 [5.0] Intervention‐2: 10.0 [10.0] PES: Intervention‐1: 10.0 [5.5] Intervention‐2: 8.5 [6.7]	Intra‐oral photographs	1Y FU 3Y FU 5Y FU
11	Felice et al. 2015/Italy	Unstandarized question (1/5)	Gum esthetics	Intervention‐1: 25 pat = 5 Intervention‐2: 25 pat‐5	PES: Intervention‐1: 12.2 [1.1] Intervention‐2: 12.7 [1.0]	Intra‐oral photographs	1Y FU
12	Felice et al. 2011; Esposito et al. 2015/Italy	Unstandarized question (1/5)	Gum esthetics	Intervention‐1: 52 pat = 5 Intervention‐2: 52 pat‐5 99 pat = 5; 2 pat = 4	PES: Intervention‐1: 12.6 [1.0] Intervention‐2: 12.7 [1.2] PES: Intervention‐1: 13.0 [1.5] Intervention‐2: 12.8 [1.4]	Intra‐oral photographs	4M FY 1Y FU
13	Gallucci et al. 2011/Switzerland	VAS (0/10 cm)	Esthetics satisfaction	Intervention‐1: 91.8 [5.9]; Intervention‐2: 91.7 [10.0]	PES/WES: Intervention‐1: 13.8 [2.1]; Intervention‐2: 13.1 [2.6]	Intra‐oral photographs	2Y FU
14	Garcia‐Sanchez et al. 2021/S	Questionnarie Bruyn et al., (1–100%) VAS (0/10 cm)	Overall satisfaction Overall satisfaction	Intervention‐1: 69.2%; Intervention‐2: 84.6% NR	PES: Intervention‐1: 10.5 [2.4]; Intervention‐2: 10.5 [2.4]; WES: Intervention‐1: 6.9 [1.9]; Intervention‐2: 6.9 [2.4]	Intra‐oral photographs Intra‐oral photographs	1Y FU
15	Gjelvold et al. 2017/Swe	OHIP‐14 (0/56) VAS (0/100 mm)	Health‐related quality Overall satisfaction	OHIP‐14: Intervention‐1 18.6 [9.0]; Intervention‐2: 18.6 [5.3] OHIP‐14: Intervention‐1 16.4 [7.0]; Intervention‐2: 16.9 [4.6] OHIP‐14: Intervention‐1 15.3 [2.5]; Intervention‐2: 16.4 [3.8] VAS: Intervention‐1: 87.9 [11.3]; Intervention‐2: 89.6 [9.5]	Gingival zenith: Intervention‐1: −0.3 [−0.5]; Intervention‐2: −0.1 [0.5]; PES: Intervention‐1: 9.4 [2.9]; Intervention‐2: 8.5 [2.2]; WES: Intervention‐1: 7.0 [1.6]; Intervention‐2: 7.0 [1.4]; PES: Intervention‐1: 10.3 [2.6]; Intervention‐2: 9.7 [2.3]; WES: Intervention‐1: 7.5 [1.6]; Intervention‐2: 7.5 [1.3]; PES: Intervention‐1: 10.6 [2.3]; Intervention‐2: 10.3 [2.4]; WES: Intervention‐1: 7.8 [1.3]; Intervention‐2: 7.7 [1.3]	Superimposed photographs of study casts (ImageJ software, USA) Intra‐oral photographs Intra‐oral photographs	CI 6M FU 1Y FU
16	Hassani et al. 2021/Iran	Unstandarized question (0/4)	Overall esthetic	Intervention‐1: 16 pat = 4. yes, absolutely, 2 pat = 3. yes, partly, 1pat = 2. not sure, 1 pat = 1. not really Intervention‐2: 18 pat = 4. yes, absolutely, 1 pat = 3. yes, partly, 1pat = 2. not sure	PES: Intervention‐1: 10.2 [0.9] Intervention‐2: 11.2 [1.1] WES: Intervention‐1: 7.6 [1.2] Intervention‐2: 8.0 [1.0]	Intra‐oral photographs Intra‐oral photographs	1Y‐FU
17	Jonker et al. 2021/Netherlands	VAS (0/10 cm)	Overall satisfaction Crown satisfaction Soft tissue satisfaction	Intervention‐1: 8.3 [1.3] Intervention‐2: 8.1 [2.3] Intervention‐3: 8.1 [1.1] Intervention‐1: 9.1 [1.2] Intervention‐2: 8.6 [2.4] Intervention‐3: 9.1 [0.9] Intervention‐1: 8.5 [1.8] Intervention‐2: 8.1 [2.4] Intervention‐3: 8.1 [2.1] Intervention‐1: 8.4 [1.0] Intervention‐2: 8.4 [1.9] Intervention‐3: 7.8 [1.3] Intervention‐1: 9.0 [1.4] Intervention‐2: 8.9 [1.6] Intervention‐3: 9.0 [0.9] Intervention‐1: 8.3 [2.1] Intervention‐2: 8.4 [1.5] Intervention‐3: 8.1 [2.0] Intervention‐1: 7.9 [1.4] Intervention‐2: 8.0 [2.1] Intervention‐3: 7.9 [1.4] Intervention‐1: 9.1 [1.0] Intervention‐2: 8.8 [1.1] Intervention‐3: 9.0 [1.2] Intervention‐1: 8.3 [1.5] Intervention‐2: 8.1 [1.7] Intervention‐3: 8.0 [1.8]	PES: Intervention‐1: 6.1 [1.4] Intervention‐2: 6.2 [1.6] Intervention‐3: 6.3 [1.1] WES: Intervention‐1: 7.6 [1.4] Intervention‐2: 7.7 [1.6] Intervention‐3: 7.2 [1.5] PES: Intervention‐1: 7.0 [1.4] Intervention‐2: 6.8 [1.3] Intervention‐3: 6.8 [1.3] WES: Intervention‐1: 7.8 [1.0] Intervention‐2: 7.6 [1.5] Intervention‐3: 7.3 [1.4] PES: Intervention‐1: 7.3 [1.7] Intervention‐2: 7.1 [1.5] Intervention‐: 7.0 [1.4] WES: Intervention‐1: 8.0 [0.9] Intervention‐2: 7.8 [1.3] Intervention‐3: 7.3 [1.7]	Intra‐oral photographs Intra‐oral photographs	BL 6M FU 1Y FU
18	Jonker et al. 2018/Netherlands	VAS (0/10 cm)	Overall satisfaction Crown esthetics Mucosa estethics	Intervention‐1: 8.5 (7.7–9.4) Intervention‐2: 8.5 (8.0–9.2) Intervention‐1: 9.7 (8.5–10.0) Intervention‐2: 9.1 (8.7–9.8) Intervention‐1: 8.5 (6.9–9.0) Intervention‐2: 8.1 (6.8–9.3) Intervention‐1: 9.0 (8.1–9.7) Intervention‐2: 8.8 (8.5–9.5) Intervention‐1: 9,2 (8.0–9.8) Intervention‐2: 9.6 (8.7–9.9) Intervention‐1: 9.0 (7.6–9.6) Intervention‐2: 7.9 (7.3–9.4) Intervention‐1: 8.4 (7.8–9.7) Intervention‐2: 8.3 (7.5–9.4) Intervention‐1: 9.6 (8.5–9.9) Intervention‐2: 9.1 (8.6–9.9) Intervention‐1: 9.2 (6.6–9.9) Intervention‐2: 8.5 (7.9–9.5)	PES: Intervention‐1: 7.0 (5.5–7.8) Intervention‐2: 6.3 (5.5–7.9) WES: Intervention‐1: 9.0 (8.1–9.5) Intervention‐2: 9.0 (8.1–9.5) PES: Intervention‐1: 7.5 (7.0–9.0) Intervention‐2: 8.0 (6.5–8.6) WES: Intervention‐1: 9.0 (8.5–10.0) Intervention‐2: 9.0 (8.5–9.5) PES: Intervention‐1: 8.0 (6.5–9.5) Intervention‐2: 7.8 (7.0–9.0) WES: Intervention‐1: 9.5 (8.0–9.5) Intervention‐2: 9.5 (8.5–9.6)	Intra‐oral photographs	1M FU 6M FU 1Y FU
19	Meijndert et al. 2007/Netherlands	VAS (0/10 cm)	Overall satisfaction	8.5 (6–10)	ICAI‐crown: 1.3 (0–9) ICAI‐mucosa: 3.4 (0–11) ICAI‐total: 4.8 (0–17)	Intra‐oral photographs Intra‐oral photographs Intra‐oral photographs	1Y FU
20	Ruiz Henao et al. 2021; Henao et al. 2024/Spain	Questionnarie Bruyn et al., NRS (0/10)	Esthetic appearance General satisfaction	Intervention‐1: 9.5 (6–10.0) Intervention‐2: 10.0 (8–10.0) Intervention‐1: 10.0 (9–10.0) Intervention‐2: 10.0 (9–10.0) Intervention‐1: 9.0 (8.4–9.6) Intervention‐2: 9.3 (8.8–9.9) Intervention‐1: 9.4 (9.0–9.8) Intervention‐2: 9.6 (9.2–10.0)	Esthetic rated by clinician by NRS: Intervention‐1: 8.5 Intervention‐2: 9.0; ICAI‐crown: Intervention‐1: 3.6; Intervention‐2: 3.9 ICAI‐mucosa: Intervention‐1: 2.3; Intervention‐2: 2.3 ICAI‐total: Intervention‐1: 6.0; Intervention‐2: 6.3 PES: Intervention‐1: 7.8 [1.2] Intervention‐2: 7.8 [1.7] Esthetic rated by clinician by NRS: Intervention‐1: 7.5 Intervention‐2: 8.1 ICAI‐crown: Intervention‐1: 2.5; Intervention‐2: 2.8 ICAI‐mucosa: Intervention‐1: 1.9; Intervention‐2: 2.5 ICAI‐total: Intervention‐1: 4.5; Intervention‐2: 5.2 PES: Intervention‐1: 7.4 Intervention‐2: 7.4	Paper‐based 1‐item scale Intra‐oral photographs Intra‐oral photographs Intra‐oral photographs Intra‐oral photographs	1Y FU 5Y FU
21	Santhanakrishnan et al. 2021; Santhanakrishnan et al. 2024/India	VAS (0/10 cm)	Overall satisfaction	Intervention‐1: 9.0 [0.6] Intervention‐2: 8.7 [0.6] Intervention‐3: 8.4 [0.8] Intervention‐1: 9.2 [0.6] Intervention‐2: 8.4 [1.0] Intervention‐3: 8.0 [1.1]	PES: Intervention‐1: 10.2 [1.4] Intervention‐2: 11.7 [1.8] Intervention‐3: 11.2 [2.1] PES: Intervention‐1: 12.5 [1.0] Intervention‐2: 10.1 [1.3] Intervention‐3:9.1 [1.0]	NR	6M FU 1Y FU
22	Slagter et al. 2015; Slagter et al. 2021; Donker et al. 2024/Netherlands	VAS (0/10 cm)	Overall satisfaction	Intervention‐1: 7.9 [1.8] Intervention‐2: 8.1 [1.7] Intervention‐1: 8.4 [1.1] Intervention‐2: 8.7 [1.8] Intervention‐1: 8.6 [1.8] Intervention‐2: 8.4 [1.4]	Papilla Index *Mesial*: Intervention‐1: 2.1 [0.8]; Intervention‐2: 2.3 [0.6]; *Distal*: Intervention‐1: 2.3 [0.7]; Intervention‐2: 2.0 [0.6]; mPES: Intervention‐1: 7.8 [1.6]; Intervention‐2: 7.4 [1.5] PES/WES: Intervention‐1: 16.2 [2.2] Intervention‐2: 151 [1.7] WES: Intervention‐1: 7.9 [1.7]; Intervention‐2: 7.6 [1.0] Papilla Index *Mesial*: Intervention‐1: 2.5 [0.7]; Intervention‐2: 2.6 [0.7]; *Distal*: Intervention‐1: 2.5 [0.7]; Intervention‐2: 2.4 [0.6] mPES: Intervention‐1: 7.8 [1.6]; Intervention‐2: 7.0 [1.7] PES/WES: Intervention‐1: 15.4 [2.6] Intervention‐2: 15.7 [2.1] WES: Intervention‐1: 7.5 [2.1]; Intervention‐2: 8.2 [1.5] Papilla Index *Mesial*: Intervention‐1: 2.5 [0.7]; Intervention‐2: 2.6 [0.7]; *Distal*: Intervention‐1: 2.5 [0.7]; Intervention‐2: 2.4 [0.6] mPES: Intervention‐1: 7.8 [1.6]; Intervention‐2: 7.0 [1.7] PES/WES: Intervention‐1: 15.4 [2.6]; Intervention‐2: 15.7 [2.1] WES: Intervention‐1: 7.5 [2.1]; Intervention‐2: 8.2 [1.5]	Clinically Intra‐oral photographs Intra‐oral photographs Intra‐oral photographs	1M FU 5Y FU 10Y FU
23	Slagter et al. 2016; Slagter et al. 2021; Meijer et al. 2024/Netherlands	VAS (0/100 mm) OHIP‐14 (0/4)	Overall satisfaction 5. Self‐consciousness of teeth 13. General satisfaction Overall satisfaction	Intervention‐1: 80.7 [12.3]; Intervention‐2: 80.7 [12.3]; Intervention‐1: 82.4 (14.3) Intervention‐2: 77.7 (17.2) Intervention‐1: 15 pat = 2, 3 pat = 3, 1 pat = 4; Intervention‐2: 13 pat = 2, 4 pat = 3, 3 pat = 4; Intervention‐1: 13 pat = 2, 3 pat = 3; 1 pat = 4; 1 pat = 5; Intervention‐2: 17 pat = 2, 2 pat = 3; 1 pat = 4; Intervention‐1: 82.4 [14.3]; Intervention‐2: 77.7 [17.2] Intervention‐1: 88.5 (83.9–93.2); Intervention‐2: 80.7 (74.9–86.0)	Papilla Index *Mesial*: Intervention‐1: 1.8 [1.0]; Intervention‐2: 2.1 [0.9]; *Distal*: Intervention‐1: 1.8 [1.0]; Intervention‐2: 2.0 [0.9] PES Intervention‐1: 7.4 [1.5]; Intervention‐2: 7.8 [1.6]; WES Intervention‐1: 7.6 [1.0]; Intervention‐2: 7.9 [1.7]; Papilla Index: Intervention‐1: *Mesial*: 6 pat = 1; 5 pat = 2; 9 pat = 3; *Distal*: 7 pat = 1; 7 pat = 2; 6 pat = 3; Intervention‐2: *Mesial*: 3 pat = 1; 8 pat = 2; 9 pat = 3; *Distal*: 3 pat = 1; 8 pat = 2; 9 pat = 3; PES: Intervention‐1: 7.4 [1.5] Intervention‐2: 7.5 [1.6] WES: Intervention‐1: 7.9 [1.1] Intervention‐2: 8.1 [0.9] Papilla Index *Mesial*: Intervention‐1: 2.4 [0.7]; Intervention‐2: 2.2 [0.8]; *Distal*: Intervention‐1: 2.3 [0.7]; Intervention‐2: 2.2 [0.8]; PES: Intervention‐1: 7.5 [1.3]; Intervention‐2: 7.4 [1.8]; WES: Intervention‐1: 7.0 [1.3]; Intervention‐2: 7.3 [1.1]; Papilla Index *Mesial*: Intervention‐1: 2.0; Intervention‐2: 3.0; *Distal*: Intervention‐1: 2.0; Intervention‐2: 2.0; PES: Intervention‐1: 6.9; Intervention‐2: 7.4; PES/WES: Intervention‐1: 14.0; Intervention‐2: 15.0; WES: Intervention‐1: 7.1; Intervention‐2: 7.6	Clinically Intra‐oral photographs Intra‐oral photographs	1M FU 1Y FU 5Y FU 10Y FU
24	Vazouras et al. 2022/USA	VAS (0/10 cm)	Color of tooth Color of gum Shape of gum Overall smile	Intervention‐1: 7.1 [1.6] Intervention‐2: 8.1 [1.4]; Intervention3: 8.7 [0.9] Intervention‐1: 7.0 [1.7] Intervention‐2: 8.1 [1.3]; Intervention‐3: 8.8 [1.0] Intervention‐1: 7.8 [1.4] Intervention‐2: 8.4 [1.4]; Intervention3: 8.2 [1.3] Intervention‐1: 7.6 [1.7] Intervention‐2: 8.5 [1.3]; Intervention‐3: 8.6 [1.2] Intervention‐2: 9.0 [1.4] Intervention‐3: 9.1 [1.0] Intervention‐2: 9.3 [1.0] Intervention‐3: 9.1 [1.1] Intervention‐2: 8.8 [1.5] Intervention‐3: 9.1 [0.9] Intervention‐2: 9.0 [1.4] Intervention‐3: 9.2 [0.9]	PES: Intervention‐1: 9.6 [1.4] Intervention‐2: 10.1 [1.1] Intervention‐3: 10.8 [0.8] PES: Intervention‐1: NR Intervention‐2: 11.2 [1.2] Intervention‐3: 12.0 [0.7]	NR	BL 1Y FU
25	Wanis et al. 2022/Egypt	Unstandarized questions (1/10)	Implant compared to natural tooth Color of implant crown compared to natural tooth Gingival contour compared to natural tooth Gingival color compared to natural tooth General satisfaction	Intervention‐1: 7.5 [0.5]; Intervention‐2: 7.8 [0.7]; Intervention‐1: 7.2 [0.7]; Intervention‐2: 7.3 [0.5]; Intervention‐1: 8.3 [0.6]; Intervention‐2: 8.3 [0.6]; Intervention‐1: 10.0 [0.0]; Intervention‐2: 10.0 [0.0]; Intervention‐1: 9.0 [0.6]; Intervention‐2: 9.1 [0.6]	Mid‐facial recession: Intervention‐1 0.4 [0.4]; Intervention‐2 0.2 [0.3] PES: Intervention‐1 10.8 [1.5]; Intervention‐2 11.3 [1.6]	Clinically NR	1Y FU
26	Wittneben et al., 2023/Switzerland	VAS (0/100 mm)	Overall satisfaction Crown Esthetics Mucosa Estethics	Intervention‐1: 93.1 [8.7]; Intervention‐2: 91.1 [8.7]; Intervention‐1: 89.9 [13.6]; Intervention‐2: 94.0 [6.4]; Intervention‐1: 89.6 [12.8]; Intervention‐2: 87.9 [12.3]; Intervention‐1: 93.4 [15.6]; Intervention‐2: 97.2 [4.9]; Intervention‐1: 88.7 [21.1]; Intervention‐2: 92.8 [12.0]; Intervention‐1: 94.4 [12.8]; Intervention‐2: 90.7 [9.4]	ICAI: values NR mPES: values NR PES/WES: values NR WES: values NR	NR	1 week after crown insertion 5Y FU
27	Zamora et al. 2024/Spain^a^	VAS (0/10 cm)	Esthetics satisfaction	Intervention‐1: 9.0; Intervention‐2: 10.0; Intervention‐3: 8.0 Intervention‐1: 9.0; Intervention‐2: 10.0; Intervention‐3: 8.0	PES: Intervention‐1: 6.0; Intervention‐2: 7.0; Intervention3: 8.0; WES: Intervention‐1: 6.0; Intervention‐2: 8.0; Intervention‐3: 7.0; PES: Intervention‐1: 7.0; Intervention‐2: 8.0; Intervention‐3: 8.0; WES: Intervention‐1: 6.0; Intervention‐2: 8.0; Intervention‐3: 7.0	Intra‐oral photographs Intra‐oral photographs	Crown insertion 1Y FU
28	Zuiderveld et al. 2018a/Netherlands	OHIP‐14 (answered on a VAS scale) (0/10 cm) VAS (0/10 cm)	Implant and implant crown Color of crown Form of crown Color of peri‐implant mucosa Form of peri‐implant mucosa Overall satisfaction	Intervention‐1:8.7 (8.3–9.5) Intervention‐ 2: 8.4 (6.9–9.2) Intervention‐3: 9.3 (8.8–10.0) Intervention‐1:9.3 (7.2–9.9) Intervention‐2: 8.8 (6.8–9.9) Intervention‐3: 9.6 (8.9–10.0) Intervention‐1:9.0 (7.7–9.7) Intervention‐2: 9.3 (7.1–9.9) Intervention‐3: 9.8 (9.2–10.0) Intervention‐1:8.0 (6.9–9.7) Intervention‐2: 8.6 (7.2–9.6) Intervention‐3: 9.5 (8.6–10.0) Intervention‐1:7.6 (5.3–9.5) Intervention‐ 2: 8.3 (5.6–9.7) Intervention‐3: 9.0 (7.0–10.0) Intervention‐1:8.2 (7.4–8.8) Intervention‐ 2: 7.9 (6.8–9.0) Intervention‐3: 8.8 (7.6–9.6)	Mid‐buccal mucosa level Intervention‐1: −0.4 [1.5] Intervention‐2: 0.0 [1.1]; Intervention‐3: −0.1 [1.3] Papilla Index: Intervention‐1: Mesial: 9 pat = 2; 11 pat = 3; Distal: 11 pat = 2; 9 pat = 3; Intervention‐2: Mesial: 3 pat = 2; 17 pat = 3; Distal: 5 pat = 2; 15 pat = 3; Intervention‐3: Mesial: 2 pat = 1; 7 pat = 2; 11 pat = 3; Distal: 2 pat = 1; 10 pat = 2; 8 pat = 3; PES: Intervention‐1: 6.6 [1.5] Intervention‐2: 7.0 [2.4] Intervention‐2: 6.1 [1.7] WES: Intervention‐1: 8.7 [0.9] Control 2: 8.9 [1.2] Intervention‐2: 8.3 [1.6]	Intra‐oral photographs callibrated by a periodontal probe Clinically Intra‐oral photographs Intra‐oral photographs	1Y FU
29	Zuiderveld et al. 2018b; Zuiderveld et al. 2024/Netherlands	OHIP‐14 (0/56) VAS (0/10 cm)	Color of the crown Form of the crown Color of the mucosa around the crown Form of the mucosa around the crown Overall satisfaction Overall satisfaction	OHIP: Intervention‐1 10.0; Intervention‐2: 10.0 VAS: Intervention‐1 9.5; Intervention‐2: 9.3; Intervention‐1 9.3; Intervention‐2: 9.2; Intervention‐1 9.3; Intervention‐2: 9.0; Intervention‐1 8.9; Intervention‐2: 9.2; Intervention‐1: 9.2; Intervention‐2: 9.1; OHIP: Intervention‐1 6.0; Intervention‐2: 10.0 VAS: Intervention‐1 9.6; Intervention‐2: 9.4; Intervention‐1 9.3; Intervention‐2: 9.4; Intervention‐1 9.5; Intervention‐2: 9.2; Intervention‐1 9.4; Intervention‐2: 8.9; Intervention‐1: 9.2; Intervention‐2: 9.3; VAS: Intervention‐1: 8.7; Intervention‐2: 9.4	PES: Intervention‐1: 6.8 [1.5]; Intervention‐2: 6.4 [1.5]; PES/WES: Intervention‐1: 14.2 [2.4]; Intervention‐2: 13.2 [2.9]; WES: Intervention‐1: 7.4 [1.3]; Intervention‐2: 6.9 [1.9]; PES: Intervention‐1: 6.4; Intervention‐2: 6.2; PES/WES: Intervention‐1: 14.4; Intervention‐2: 13.7; WES: Intervention‐1: 8.0; Intervention‐2: 7.6	Intra‐oral photographs Intra‐oral photographs Intra‐oral photographs	BL 1M FU 1Y FU 5Y FU

Abbreviations: BL, baseline; CI, crown insertion; ClinROs, clinician‐reported outcomes; FU, follow‐up; ICAI, implant crown esthetic index; IOS, intra‐oral scan; M, month(s); mPES, modified pink esthetic score; NR, not reported; NRS, numerical rating scale; OHIP, oral health impact profile; pat, patient; PES, pink esthetic score; PROMs, patient reported outcome measurements; RCT, randomized controlled trial; VAS, visual analogue scale; WES, white esthetic score; Y, year(s).

^a^ Data reported as a median.

### 3.4 Clinician‐Reported Outcome Measures (ClinROs) Regarding Esthetics

The identified objective ClinRO regarding esthetics in the selected literature was the following:

○ Pink esthetic score (24/29)/modified Pink esthetic score (5/29)

Pink esthetic score (PES) was assessed in all evaluated studies. The original version (7 items, score 0–14) was reported in 24 studies (Fürhauser et al. 2005). Five trials used modified PES (PES: 5 items, score 0–10) (Belser et al. 2009). Fifteen RCTs evaluated PES at different time points, and the follow‐up time ranged between 1 and 10 years.

○ White esthetic score (*n* = 15)

White esthetic score (WES) was reported in 15 RCTs (5 items, score 0–10) (Belser et al. 2009). Nine studies evaluated WES at different time points, and the follow‐up time ranged between 1 and 10 years.

○ Combined pink and white esthetic score (*n* = 5)

Combined pink and white esthetic score was evaluated in five studies (PES: 5 items, score 0–10; WES: 5 items, score 0–10) (Belser et al. 2009). It was assessed in two trials at various time points, and the follow‐up time ranged between 1 and 10 years.

○ Implant crown esthetic index (ICAI) (*n* = 5)

Implant crown esthetic index was assessed in five articles (9 items, score 0–45) (Meijer et al. 2005). Four trials reported results separately regarding crown and mucosal domains. Three RCTs evaluated ICAI at different time points, and the follow‐up time ranged between 1 month and 5 years.

○ Papilla index (*n* = 4)

Papilla index was evaluated in four studies (5 items, score 0–4) (Jemt 1999). All trials evaluated papilla index at different time points, and the follow‐up time ranged between 1 month and 10 years.

○ Mucosal scarring index (*n* = 2)

Mucosal scarring index was analysed in two articles (5 items, score 0–10) (Wessels et al. 2019). One RCT evaluated it in the follow‐up period between 3 months and 3 years.

○ Midfacial recession/mid‐buccal mucosal level (*n* = 4)

Midfacial recession or mid‐buccal mucosal levels were evaluated in four studies. One trial evaluated it in the follow‐up period between 3 months and 3 years.

○ Buccal peri‐implant mucosa changes at the zenith (*n* = 2)

Buccal peri‐implant mucosa changes at the zenith were analyzed in two trials. One study evaluated it in the follow‐up period between 6 months and 1 year.

Table 3 provides detailed qualitative information on the objective ClinROs and measuring instruments used in the selected studies. One subjective ClinRO was identified for the esthetic evaluation; this was assessed by the clinician.

### 3.5 Individual Participant Data (IPD) Results

The overall analysis was conducted at the patient level, focusing on single‐implant‐supported restorations. Data from 15 RCTs, including 700 participants, were analyzed. Fourteen studies included PES and modified PES as ClinRO measures, along with VAS and OHIP‐14 as PROMs, forming the basis of the IPD meta‐analysis and correlation assessments.

#### 3.5.1 PES and Patient Satisfaction Measured by VAS

Data from 675 patients were evaluated at different time points for PES and patient satisfaction (VAS).

- Crown insertion (baseline)

**FIGURE 3:**
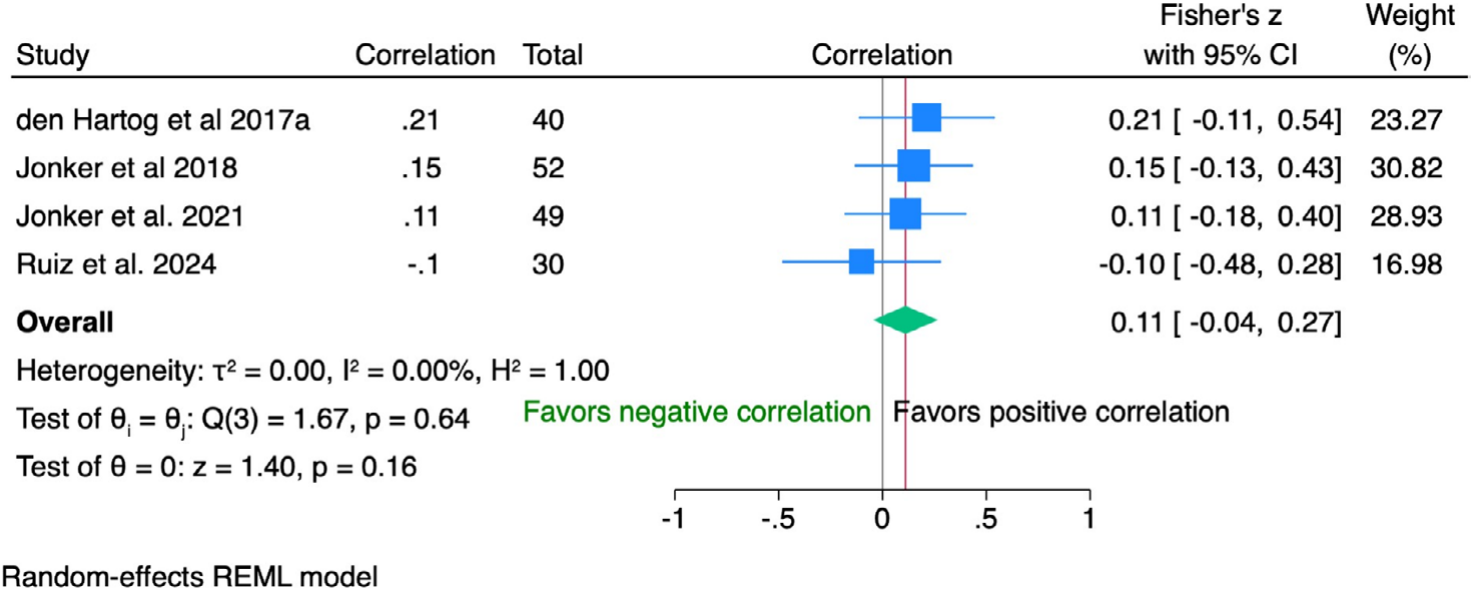
Individual patient data meta‐analysis and forest plot between patients' overall satisfaction measured by the visual analogue scale and the modified pink esthetic score (PES), measured by the clinician at crown insertion (baseline).

IPD from 171 patients across four RCTs showed a weak positive correlation (*r* = 0.11, 95% CI [−0.04; 0.27]) between PES and VAS, which was not statistically significant (*p* = 0.16). The data were homogeneous (*I* ^2^ = 0%) (Figure 3).

- 6‐month follow‐up

**FIGURE 4:**
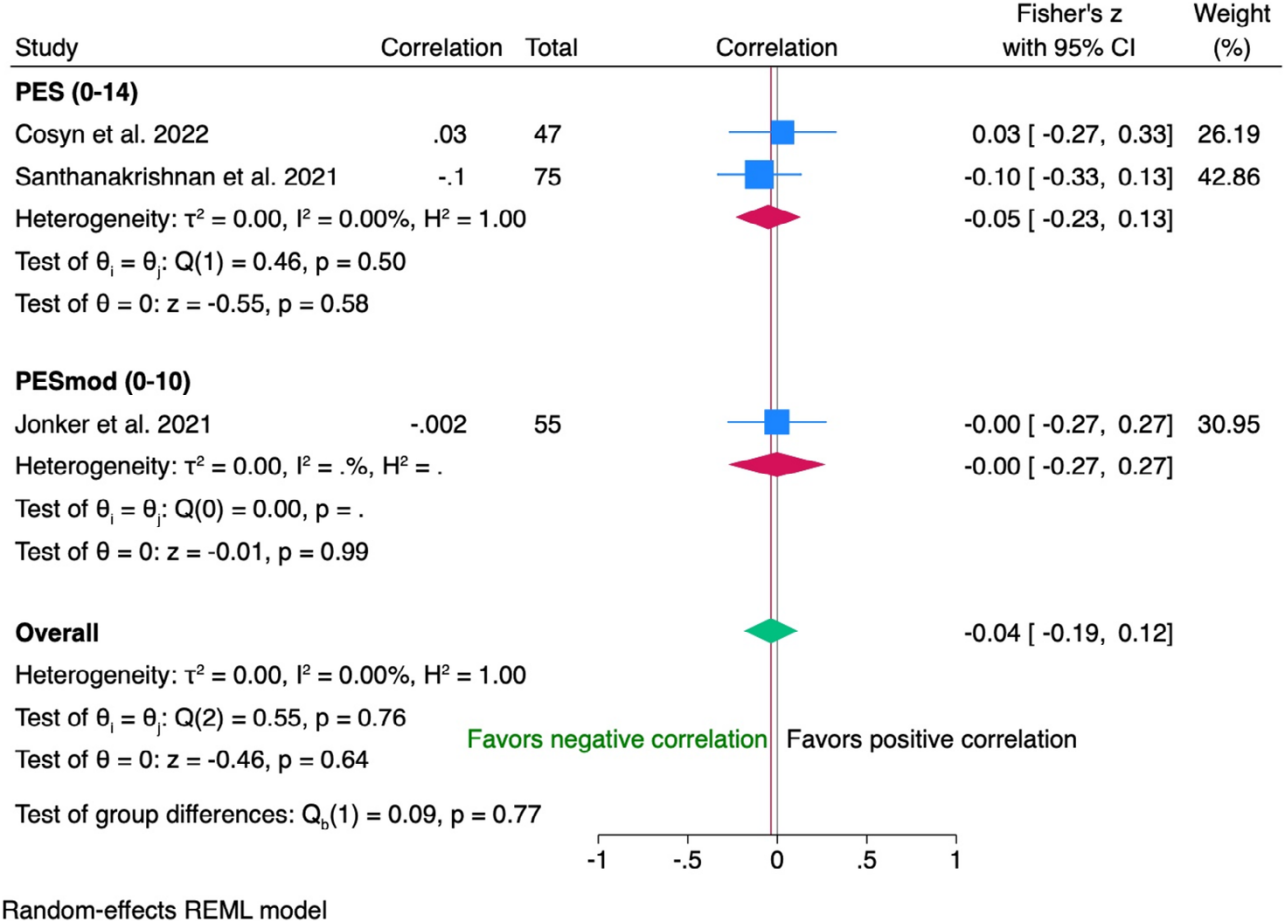
Individual patient data meta‐analysis and forest plot between patients' overall satisfaction measured by visual the analogue scale and the pink esthetic score (PES) and the modified PES, measured by the clinician at 6‐month follow‐up.

Data from 122 patients between VAS and in two studies (PES) and 55 patients in one trial (modified PES) showed a negligible negative correlation (*r* = −0.04, 95% CI [−0.19; 0.12]) (Figure 4). No heterogeneity (*I* ^2^ = 0%) or significant differences between PES and modified PES (*p* = 0.77) were observed.

- 1‐year follow‐up

**FIGURE 5:**
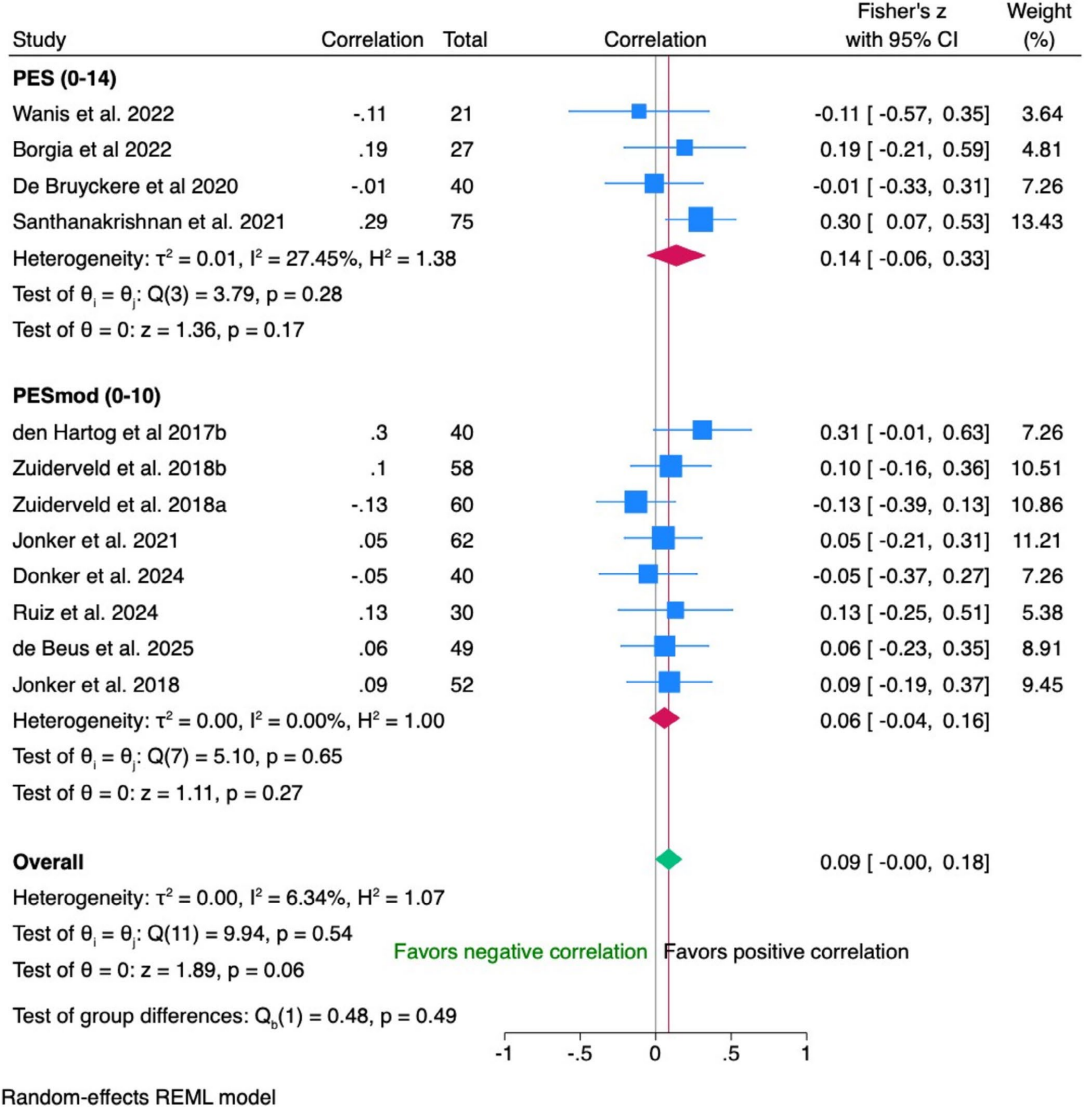
Individual patient data meta‐analysis and forest plot between patients' overall satisfaction measured by the visual analogue scale and the pink esthetic score (PES) and the modified PESm measured by the clinician at 1‐year follow‐up.

IPD from 502 patients in 11 studies showed a negligible positive correlation between PES or modified PES and VAS (*r* = 0.09) with a trend towards statistical significance (*p* = 0.06). Minimal heterogeneity (*I* ^2^ = 6.3%) was observed across studies (Figure 5).

- 5‐year follow‐up

**FIGURE 6:**
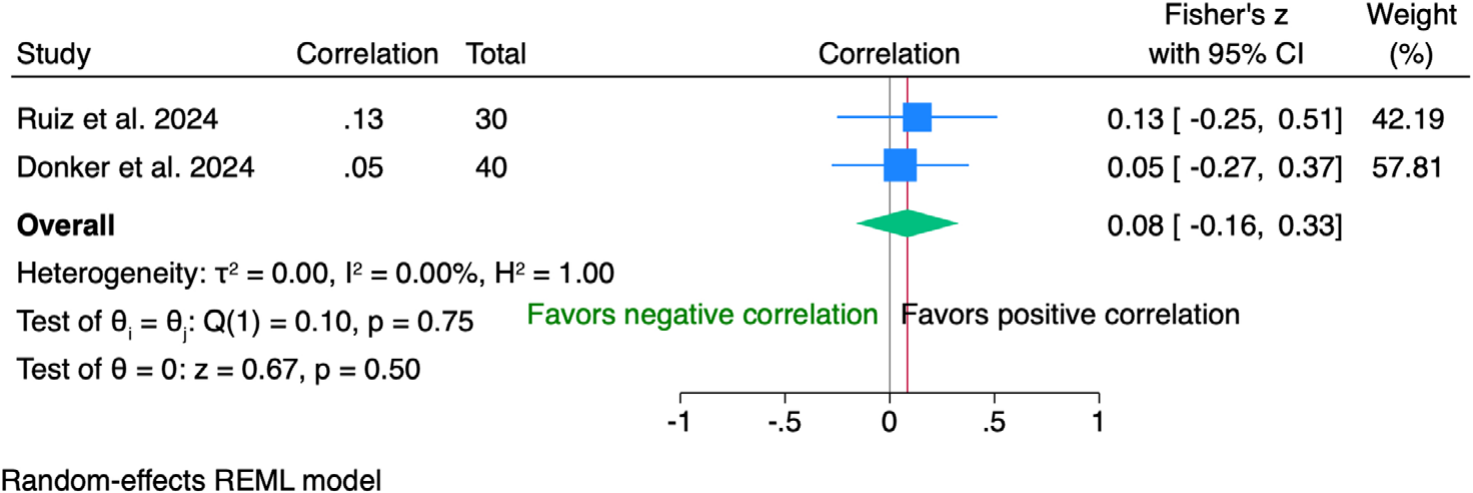
Individual patient data meta‐analysis and forest plot between patients' overall satisfaction measured by the visual analogue scale and the pink esthetic score (PES), measured by the clinician at 5‐year follow‐up.

Data from 70 patients in two trials showed no correlation (*r* = 0.08, 95% CI [−0.16; 0.33], *p* = 0.50) between PES and VAS. The findings were consistent across studies with no heterogeneity (*I* ^2^ = 0%) (Figure 6).

- 10‐year follow‐up

**FIGURE 7:**
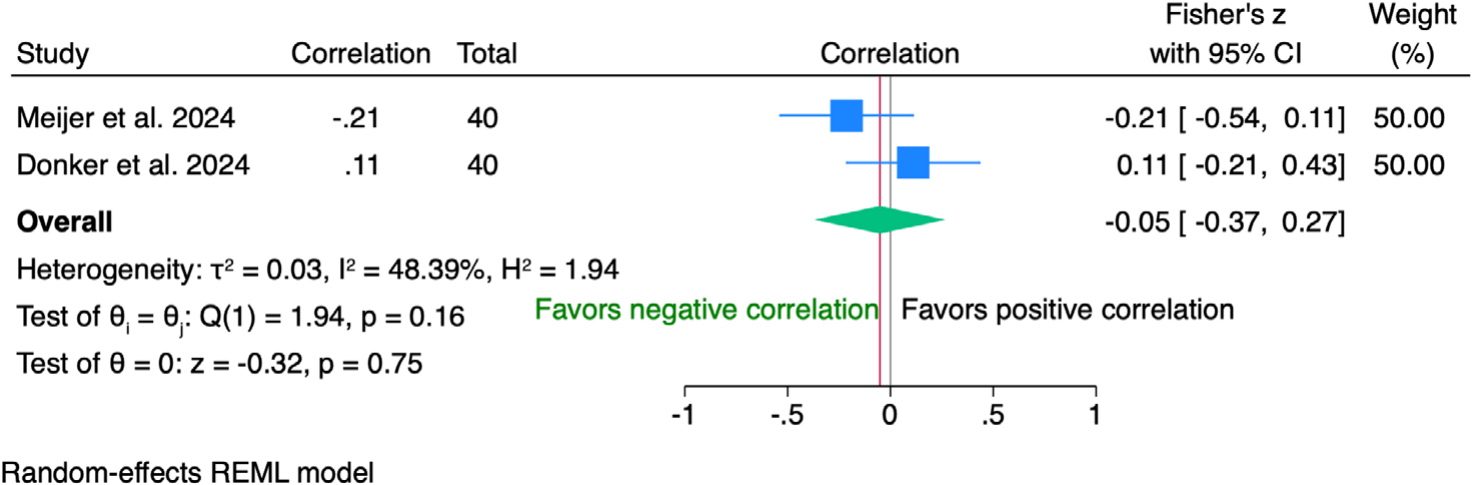
Individual patient data meta‐analysis and forest plot between patients' overall satisfaction measured by the visual analogue scale and the modified pink esthetic score (PES), measured by the clinician at 10‐year follow‐up.

Data from 80 patients in two studies showed a negligible negative correlation between modified PES and VAS (*r* = −0.05, 95% CI [−0.37; 0.27], *p* = 0.75) (Figure 7). Moderate heterogeneity (*I* ^2^ = 48.39%) suggested variability between studies, with concerns about bias due to dropout rates according to the risk of bias assessment.

#### 3.5.2 WES and Patient Satisfaction Measured by VAS

- 1‐year follow‐Up

**FIGURE 8:**
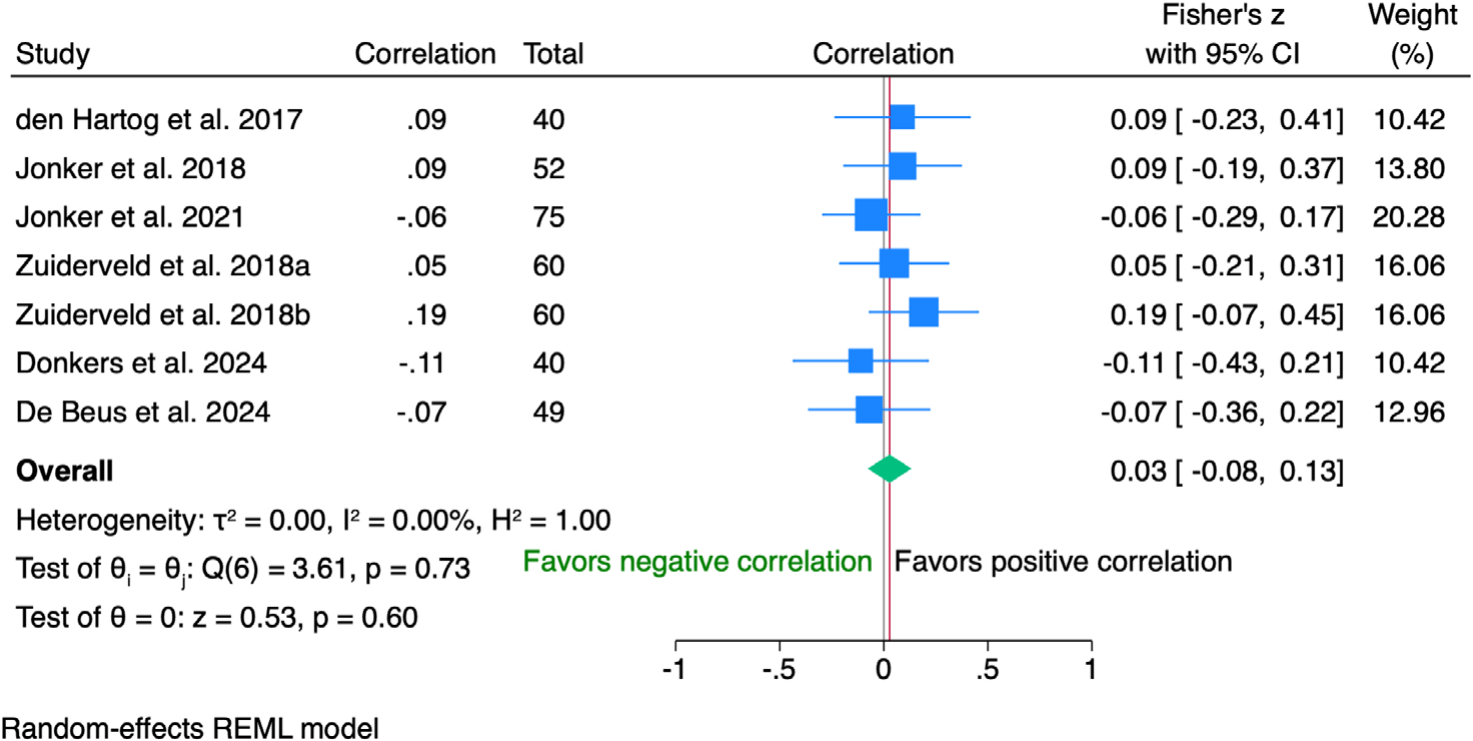
Individual patient data meta‐analysis and forest plot between patients' overall satisfaction measured by the visual analogue scale and the white esthetic score (WES), measured by the clinician at 1‐year follow‐up.

Data from 376 patients in seven studies showed a negligible positive correlation between WES and patient satisfaction measured by VAS (*r* = 0.03, 95% CI [−0.08; 0.13], *p* = 0.60), with no heterogeneity (Figure 8).

- 10‐year follow‐Up

**FIGURE 9:**
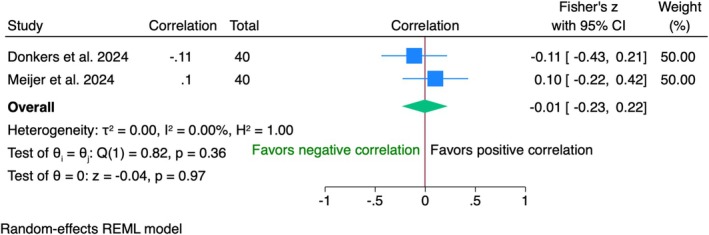
Individual patient data meta‐analysis and forest plot between patients' overall satisfaction measured by the visual analogue scale and the white esthetic score (WES), measured by the clinician at 10‐year follow‐up.

Data from 80 patients in two trials showed no significant correlations (*r* = −0.01, 95% CI [−0.23; 0.22], *p* = 0.97) between VAS and WES (Figure 9). No heterogeneity was found; though concerns about dropout‐related bias exist.

#### 3.5.3 PES and OHIP‐14 (1‐Year Follow‐Up*)*

**FIGURE 10:**
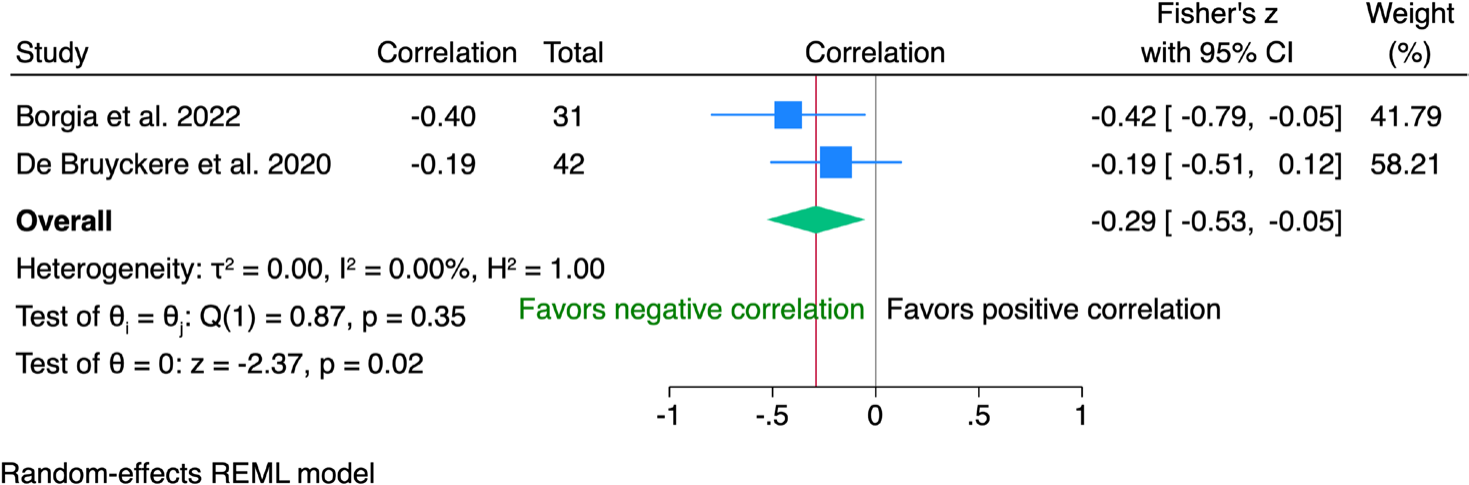
Individual patient data meta‐analysis and forest plot between the oral health impact profile (OHIP) questionnaire and the pink esthetic score (PES), measured by the clinician at 1‐year follow‐up.

Data from 73 patients in two studies revealed a significant weak negative correlation (*r* = −0.29, 95% CI [−0.53; 0.05], *p* = 0.02) between PES and patient satisfaction measured by OHIP‐14 (Figure 10). No heterogeneity was detected (*I* ^2^ = 0%).

To sum up, across multiple time points, no strong or consistent correlation was found between clinician‐reported esthetic outcomes (PES/WES) and patient‐reported satisfaction (VAS/OHIP‐14). Significant findings were limited, with concerns about bias and variability between studies.

## 4 Discussion

The present systematic review on single implant‐supported crowns in the esthetic zone primarily revealed: (i) No correlation between patient satisfaction using VAS and PES during different follow‐ups up to 10 years; (ii) a significant negative correlation between PES and OHIP‐14 questionnaires.

This study used an individual participant data (IPD) meta‐analysis of RCTs, a specific type of systematic review, designed to address a focused clinical question. Unlike traditional meta‐analyses, which rely on aggregated data, IPD involves collecting, checking and reanalysing the raw individual‐level data from multiple related studies. This approach allows for a more precise exploration of differential treatment effects across patients (Riley et al. 2010).

In this systematic review, IPD allowed for a more detailed exploration of individual variations in esthetics perceptions and patient satisfaction. For example, patients with similar PES scores (e.g., PES = 13) may report varying satisfaction levels, ranging from 60 mm to 90 mm on VAS scales. Traditional aggregated data meta‐analysis often masks these individual variations, potentially leading to oversimplified conclusions regarding the relationship between patient and clinician‐reported outcomes in esthetics (Veroniki et al. 2016). We were able to include IPD from 14 out of 29 eligible RCTs, comprising 48% of all known randomised participants. The incorporation of updated follow‐up data from some trials allowed for a more precise analysis of the potential association between patient‐ and clinician‐reported esthetic outcomes.

A previously published systematic review evaluated methods of assessment and interventions in the context of implant therapy. It highlighted that PROs were often underreported, and they needed to be incorporated as a key methodological element in future clinical research (Avila‐Ortiz et al. 2023). Their importance was also mentioned in the Core Outcome Set in implant dentistry (Tonetti et al. 2023). The present review found low heterogeneity among included studies, with most of the trials conducted in university settings, with an increasing trend of PROMs assessment in more recent publications (Thoma et al. 2024). Several studies even evaluated PROMs as primary outcomes, aligning with findings from another review that observed a growing interest in assessing esthetic outcomes using both PROMs and objective indices in implant‐related dentistry (De Bruyn et al. 2015). In daily practice, the association between patients and clinicians' perspectives is an important factor for decision‐making and evaluation of outcomes.

Despite extensive analysis, no correlation was found between PROs and ClinROs regarding esthetics of single implant‐supported crowns in this systematic review. Specifically, there was no significant correlation between patient satisfaction, measured by VAS, and PES (ClinRO) across follow‐ups up to 10 years. Several factors may explain the lack of correlation. First, the available raw data for IPD were limited in sample size and varied in follow‐up periods, which may have masked correlations. Second, current esthetic indices may not fully capture parameters that are important to patients, focusing instead on aspects that are less relevant to them. Third, clinicians and patients may prioritize different esthetic factors. Patients tend to focus on overall appearance and harmony, whereas dentists may focus more on the surrounding soft tissues, which are central to the PES assessment (Cosyn, Eeckhout, et al. 2021; Mancini et al. 2023). Furthermore, soft tissue augmentations that influence clinician evaluations may be less relevant to patients' perceptions (Thoma et al. 2023, Ramanuskaite et al. 2025).

In recent years, WES has become a widely used index for assessing the esthetic of single implant restorations. However, several studies have questioned its reliability compared to other esthetic indices (Cosyn, Wessels, et al. 2021; Hof et al. 2018). In this systematic review, seven articles reported WES, and no correlation between WES and patient esthetic satisfaction was found, further underscoring the discrepancies between ClinROs and patient perception. This highlights a gap between clinician assessments and patient perceptions, which should be considered when planning and outcome evaluation.

Patient satisfaction with treatment results seems to be influenced by factors beyond esthetic outcomes alone (Fava et al. 2015). A key limitation of some included studies is the lack of blinded investigators in outcome assessments, which may introduce bias, as clinicians evaluating the outcomes often performed the treatment (e.g., positive judgement, professional relationship between patient and clinician). Moreover, ensuring patients that their responses remain confidential is essential to encourage honest and accurate reporting (Wittich et al. 2024). Clear instructions from independent investigators are essential for obtaining reliable PROs.

Different methods, timing tools and scale are used to evaluate PROs in implant‐related dentistry. Historically, non‐standardized paper questionnaires with Likert scales, VAS and open questions were used (Cosyn et al. 2017). While patient interviews and non‐standardized questionnaires provide valuable exploratory data, their validity is limited. Consequently, comparing studies using such measures is challenging and may yield unreliable conclusions. Currently, the “Oral Health Related Quality of Life” (OHRQoL) and “Oral Health Impact Profile” (OHIP) are the only validated questionnaires used in implant dentistry. The original version of OHIP consists of 49 questions evaluating seven core areas and the shorter versions consist of 14, 20, or 21 questions. However, both standardized questionaries primarily evaluate physical and psychological pain, functional limitations and overall quality of life rather than esthetics.

In this review, a significant negative correlation was observed when patient satisfaction was measured using OHIP‐14. This inverse relationship highlights the complex interaction between specialized esthetic indices and health‐related quality of life factors reported by patients. There is a clear demand to develop specifically validated questionnaires for esthetic assessments in implant dentistry.

As patient‐reported outcomes become an integral part of esthetic implant treatment planning, their assessment must be standardized and anonymized to ensure they are a central component of daily practice. Notably, 10 out of 42 articles raised some concerns related to outcome measurement methods. The overall strength of evidence was rated as moderate according to GRADE guidelines (Schunemann et al. 2008). Despite its limitations, this systematic review provides relevant information into the utilization of PROs in relation to ClinROs for the esthetics of implant‐supported restorations.

The present review has limitations. First, there was heterogeneity among PROs and ClinROs tools used to assess patient perception. Secondly, this review did not focus on treatment efficacy. Furthermore, by including only RCTs, valuable data from high‐quality non‐randomized studies might have been excluded. Additionally, obtaining raw data from all eligible studies was challenging, with limited responses from corresponding authors. Lastly, not all the studies provided detailed information on how PROs were assessed.

From a clinician's perspective, the results of this IPD meta‐analysis are frustrating. Considering all the efforts in terms of tissue regeneration (encompassing morbidity, time and costs for patients) and prosthetic adjustments (encompassing provisional crowns, several try‐in sessions), the outcomes of this analysis are in strong contrast to the philosophy of how patients are informed and treated. It remains unanswered whether these surgical and prosthetic steps remain masked behind deficiencies in assessment, or whether the population is rather overtreated as a whole.

## 5 Conclusions

PES and WES showed no correlation with patient satisfaction, as measured by VAS and OHIP‐14, in terms of esthetics for up to 10 years after final crown placement.

## Author Contributions

**Sofya Sadilina:** data curation, project administration, investigation, writing – original draft. **Nicolas P. A. Müller:** investigation, data curation, writing – review and editing. **Franz J. Strauss:** formal analysis, validation, visualization, writing – review and editing. **Ronald E. Jung:** conceptualization, funding acquisition, software, writing – review and editing. **Daniel S. Thoma:** conceptualization, methodology, resources, writing – review and editing. **Stefan P. Bienz:** conceptualization, methodology, resources, supervision, writing – original draft, writing – review and editing.

## Ethics Statement

The authors have nothing to report.

## Conflicts of Interest

The authors declare no conflicts of interest.

## Supporting information


**Data S1:** clr70019‐sup‐0001‐supinfo.docx.

## Data Availability

The datasets used in the study are available from the corresponding author upon reasonable request.
