# Supplementary material for: Patient‐Reported and Clinician‐Reported Esthetic Outcomes at Implant Sites Are Not Associated: A Systematic Review With Individual Participant Data Meta‐Analysis
Source: Clin Oral Implants Res. 2025 Aug 20;36(11):1382–411. doi: 10.1111/clr.70019 (PMC12598916; doi:10.1111/clr.70019)
Supplement: Supplementary file 1 — Data S1: clr70019‐sup‐0001‐supinfo.docx. [file CLR-36-1382-s001.docx]

**Table S1.** Search strategies.

| Databases | |
| --- | --- |
| Medline  (via PUBMED) | #1 "Dental Implants" [MeSH terms] OR "Dental Implantation" [MeSH terms] OR "Dental Prosthesis, Implant-Supported" [MeSH terms] OR "Implant, Implant Supported" [Title/Abstract]  #2 “Visual Analog Scale” [MeSH terms] OR Quality of Life [All Fields] OR Patient Satisfaction [All Fields] OR Patient Reported Outcome Measure* [All Fields] OR "PROM*" [All Fields] OR "VAS" [All Fields] OR "Numerical Rating Scale" [All Fields] OR "OHIP" [All Fields] OR Questionnaire* [All Fields] OR Patient Expectation [Title/Abstract] OR Patient Centered Outcome OR Patient Opinion OR Patient-Reported OR Patient Related  #3 "Esthetics, Dental" [MeSH terms] OR Operator Reported Outcome Measure* [All Fields] OR “Clinician Reported Outcome*” [All Fields] OR Pink Esthetic Score* [All Fields] OR PES [All Fields] OR White Esthetic Score* [All Fields] OR WES [All Fields] OR Esthetic Outcome* [All Fields] OR Esthetic Index [All Fields] OR VAS [All Fields] OR “Visual Analog Scale” [MeSH terms] OR Aesthet* [All Fields]  #4 #1 AND #2 AND #3 |
| Embase | #1 'tooth implant'/exp OR ‘Tooth implantation’/exp OR ‘Implant-supported denture’/exp OR ‘Implant, Implant Supported’  #2 ‘visual analog scale’/exp OR 'quality of life' OR patient reported outcome measure OR patient satisfaction OR prom* OR vas OR ‘numerical rating scale’ OR ohip OR questionnaire OR 'patient centered outcome' OR 'patient-reported' OR ‘patient related’  #3 'dental procedure' OR 'operator reported outcome measure*' OR 'clinician reported outcome*' OR 'pink esthetic score' OR pes OR 'white esthetic score' OR wes OR 'esthetic outcome*' OR 'esthetic index' OR 'visual analog scale' OR aesthet*  #4 #1 AND #2 AND #3 |
| Web of Science | **#1 TS=(“Dental Implant*” OR “Dental Implantation” OR “Implant Supported” OR “oral implant*”)**  **#2** TS=(“quality of life” OR “patient satisfaction” OR “patient reported outcome measure*” OR “prom*” OR “vas” OR “visual analog scale*” OR “OHIP” OR “numerical rating scale” OR “questionnaire*” OR “patient centered outcome*” OR “patient opinion*” OR “patient perception*” OR “patient reported” OR “patient related”)  **#3** TS=(Esthetic Dental OR “Operator Reported Outcome Measure*” OR “Clinician Reported Outcome*” OR “Pink Esthetic Score*” OR “PES” OR “White Esthetic Score*” OR “WES” OR “Esthetic Outcome*” OR “Esthetic Index” OR “VAS” OR “Visual Analog Scale*” OR “Aesthet*”)  **#4 #1 AND #2 AND #3** |
| Register | |
| Cochrane Central  Register of Controlled  Trials (CENTRAL) | **#1** [mh "Dental Implants" ] OR [mh "Dental Implantation" ] OR [mh "Dental Prosthesis, Implant-Supported" ] OR 'implant, implant-supported'  **#2** [mh "Visual Analog Scale" ] OR " life quality" OR 'patient satisfaction' OR 'patient reported outcome measure' OR prom* OR ‘numerical rating scale’ OR OHIP OR ‘numerical rating scale’ OR Questionnaire* OR 'OR Patient Expectation' OR ‘Patient-Reported’ OR ‘Patient Centered Outcome’ OR Patient Related  **#3** [mh "Esthetics, Dental" ] OR [mh "Visual Analog Scale" ] OR Operator Reported Outcome Measure* OR ‘Clinician Reported Outcome*’ OR ‘Pink Esthetic Score*’ OR PES OR ‘White Esthetic Score*’ OR WES OR 'esthetic outcome*' OR 'esthetic index' OR VAS OR aesthet*  **#4** #1 AND #2 AND #3 |

MeSH, Medical Subject Headings.

**Table S2.** Excluded studies after full texts analysis and reasons for exclusion.

| **Study** | **Reason for Exclusion** |
| --- | --- |
| **Articles** | |
| Abd el Aziz et al., 2021 | 2 |
| Abduo et al., 2017 | 1 |
| Abduo et al., 2021 | 1 |
| Abolhasani et al., 2021 | 1 |
| Afrashtehfar, 2021 | 1 |
| Afrashtehfar et al., 2021 | 5 |
| Al-Dosari et al., 2016 | 5 |
| Alberga et al., 2019 | 5 |
| Alberga et al., 2020 | 5 |
| Allen et al., 2017 | 5 |
| Aloy-Prosper et al., 2016 | 1 |
| Aloy-Prosper et al., 2018 | 1 |
| Altay et al., 2019 | 5 |
| Amorfini et al., 2023 | 5 |
| Andersson et al., 2013 | 1 |
| Angkaew et al., 2017 | 5 |
| Antonious et al., 2021 | 1 |
| Amer et al., 2024 | 3 |
| Arora & Ivanovski, 2018b | 5 |
| Arora & Ivanovski, 2018a | 5 |
| Attia et al., 2019 | 1 |
| Azaripour et al., 2023 | 1 |
| Baer et al., 2022 | 5 |
| Baer et al., 2013 | 1 |
| Baghel et al., 2023 | 1 |
| Baldi et al., 2020 | 1 |
| Barausse et al., 2020 | 1 |
| Barausse et al., 2019 | 1 |
| Barone et al., 2016 | 1 |
| Barwacz et al., 2021 | 1 |
| Beekmans et al., 2017 | 5 |
| Beekmans et al., 2018 | 6 |
| Bin et al., 2020 | 6 |
| Boardman et al., 2016 | 5 |
| Bonde et al., 2013 | 1 |
| Bonino et al., 2018 | 1 |
| Boon et al., 2020 | 1 |
| Bose et al., 2020 | 1 |
| Branzén et al., 2015 | 5 |
| Bressan et al., 2017 | 1 |
| Cacaci et al., 2019 | 1 |
| Cairo et al., 2017 | 1 |
| Cappare et al., 2021 | 1 |
| Chang & Wennström, 2013 | 5 |
| Checchi et al., 2017 | 1 |
| Chen et al., 2022 | 5 |
| Cosyn & De Rouck, 2009 | 5 |
| Cosyn et al., 2012 | 5 |
| Cosyn et al., 2013 | 5 |
| Covani et al., 2007 | 2 |
| D'Avenia et al., 2020 | 1 |
| Dai et al., 2021 | 5 |
| De Angelis et al., 2023 | 1 |
| De Angelis et al., 2011 | 1 |
| De Rouck et al., 2008 | 5 |
| Delize et al., 2019 | 1 |
| Edinger & Beuer, 2021 | 1 |
| El Ebiary et al., 2023 | 2 |
| Ellithy et al., 2024 | 2 |
| Esposito et al., 2020 | 1 |
| Esposito, Bressan, et al., 2017 | 1 |
| Esposito, Cardaropoli, et al., 2018 | 1 |
| Esposito, Grufferty, et al., 2018 | 1 |
| Esposito, Zucchelli, et al., 2017 | 1 |
| Fabbri et al., 2017 | 1 |
| Fava et al., 2015 | 5 |
| Felice et al., 2020 | 1 |
| Felice et al., 2016 | 1 |
| Fenner et al., 2016 | 5 |
| Ferreira et al., 2023 | 5 |
| Fonseca et al., 2021 | 1 |
| Foong et al., 2022 | 1 |
| Fu et al., 2021 | 1 |
| Fügl et al., 2017 | 5 |
| Fürhauser et al., 2022 | 1 |
| García-Minguillán et al., 2020 | 1 |
| Gjelvold et al., 2017 | 1 |
| Gjelvold et al., 2020 | 5 |
| Groenendijk et al., 2021 | 5 |
| Hamdy et al., 2024 | 4 |
| Han et al., 2021 | 5 |
| Hartlev et al., 2014 | 5 |
| Hashemi et al., 2022 | 1 |
| Heinemann et al., 2016 | 1 |
| Heydecke et al., 2019 | 5 |
| Hof et al., 2015 | 5 |
| Hof et al., 2013 | 5 |
| Hof et al., 2014 | 5 |
| Hof et al., 2018 | 2 |
| Hosseini et al., 2022 | 1 |
| Hosseini et al., 2011 | 1 |
| Hosseini et al., 2013 | 1 |
| Huber et al., 2018 | 5 |
| Hurtgen et al., 2023 | 5 |
| Huynh-Ba et al., 2019 | 1 |
| Huynh-Ba et al., 2016 | 1 |
| Joda et al., 2018 | 1 |
| Jonker et al., 2020 | 5 |
| Josefsson & Lindsten, 2019 | 5 |
| Kolinski et al., 2014 | 1 |
| Kotsailidi et al., 2022 | 1 |
| Kourkouta et al., 2009 | 1 |
| Krawiec et al., 2021 | 3 |
| Kuchler et al., 2016 | 1 |
| Kunavisarut et al., 2020 | 1 |
| Kunavisarut et al., 2022 | 1 |
| Kumar et al., 2024 | 4 |
| Lambert et al., 2020 | 1 |
| Landes et al., 2012 | 5 |
| Levine et al., 2022 | 5 |
| Li et al., 2023 | 4 |
| Li et al., 2023 | 6 |
| Li Manni et al., 2020 | 1 |
| Li et al., 2019 | 5 |
| Liu et al., 2019 | 5 |
| Lorenz et al., 2019 | 1 |
| Luo et al., 2022 | 1 |
| Meijndert et al., 2020 | 5 |
| Meijndert et al., 2022 | 5 |
| Merli et al., 2018 | 1 |
| Missinne et al., 2021 | 1 |
| Momberger et al., 2022 | 5 |
| Nejatidanesh et al., 2016 | 1 |
| Nielsen et al., 2022 | 1 |
| Papi et al., 2021 | 5 |
| Pascoal et al., 2024 | 5 |
| Patil et al., 2017 | 5 |
| Pellicer et al., 2024 | 3 |
| Pollini et al., 2020 | 2 |
| Raes et al., 2013 | 5 |
| Raes et al., 2018 | 5 |
| Ren et al., 2016 | 6 |
| Roccuzzo et al., 2022 | 5 |
| Rupchandani, 2021 | 5 |
| Rutkowski et al., 2022 | 1 |
| Sala et al., 2023 | 1 |
| Santhanakrishnan et al., 2021 | 4 |
| Santing et al., 2013 | 5 |
| Sauvin et al., 2022 | 5 |
| Scheyer et al., 2017 | 5 |
| Schnider et al., 2018 | 1 |
| Schropp & Isidor, 2008 | 1 |
| Shahdad et al., 2023 | 5 |
| Shadid, 2022 | 5 |
| Shrivastava et el., 2024 | 2 |
| Sicilia-Felechosa et al., 2020 | 1 |
| Sun et al., 2020 | 6 |
| Suphanantachat et al., 2012 | 5 |
| Tartaglia et al., 2011 | 1 |
| Tavelli, Majzoub, et al., 2023 | 1 |
| Tavelli, Zucchelli, et al., 2023 | 5 |
| Taylor et al., 2014 | 5 |
| Tian et al., 2017 | 6 |
| Topçu et al., 2017 | 1 |
| Trimpou et al., 2022 | 5 |
| Tymstra et al., 2010 | 5 |
| Urban et al., 2019 | 1 |
| Van Nimwegen et al., 2016 | 5 |
| Vanlıoğlu et al., 2014 | 5 |
| Vilhjálmsson et al., 2011 | 5 |
| Wang et al., 2022 | 6 |
| Wang et al., 2021 | 6 |
| Wiesner et al., 2010 | 1 |
| Wong et al., 2020 | 5 |
| Wu et al., 2020 | 6 |
| Xu et al., 2019 | 6 |
| Yan et al., 2019 | 6 |
| Zhang et al., 2022 | 5 |
| Zhu et el., 2023 | 3 |
| Zucchelli et al., 2018 | 5 |
| Zucchelli et al., 2013 | 5 |
| Zuercher, Mancini, et al., 2023 | 5 |
| Zuercher, Ioannidis, et al., 2023 | 1 |
| Zuiderveld et al., 2019 | 5 |
| **Trials** | |
| Australian New Zealand Clinical Trials Registry, 2018a | 7 |
| Australian New Zealand Clinical Trials Registry, 2018b | 7 |
| Australian New Zealand Clinical Trials Registry, 2018c | 7 |
| Australian New Zealand Clinical Trials Registry, 2018d | 7 |
| Australian New Zealand Clinical Trials Registry, 2019 | 7 |
| Clinical Trial Registry – India, 2024a | 7 |
| Clinical Trial Registry – India, 2024b | 7 |
| Clinical Trial Registry – India, 2018 | 7 |
| Clinical Trial Registry – India, 2019 | 7 |
| Clinical Trial Registry – India, 2021 | 7 |
| Clinical Trial Registry – India, 2022 | 7 |
| German Clinical Trials Register, 2024 | 7 |
| German Clinical Trials Register, 2014 | 1 |
| German Clinical Trials Register, 2015 | 1 |
| German Clinical Trials Register, 2018 | 7 |
| German Clinical Trials Register, 2020b | 7 |
| German Clinical Trials Register, 2020a | 7 |
| German Clinical Trials Register, 2021 | 7 |
| German Clinical Trials Register, 2022a | 7 |
| German Clinical Trials Register, 2022b | 7 |
| International Standard Randomized Controlled, Trial Number Registry, 2024a | 7 |
| International Standard Randomized Controlled, Trial Number Registry, 2024b | 7 |
| International Standard Randomized Controlled, Trial Number Registry, 2020a | 7 |
| International Standard Randomized Controlled, Trial Number Registry, 2020b | 8 |
| National Clinical Trial, 2024a | 7 |
| National Clinical Trial, 2024b | 7 |
| National Clinical Trial, 2024c | 7 |
| National Clinical Trial, 2024d | 7 |
| National Clinical Trial, 2024e | 7 |
| National Clinical Trial, 2019 | 1 |
| National Clinical Trial, 2020 | 7 |
| National Clinical Trial, 2021 | 7 |
| National Clinical Trial, 2005 | 8 |
| National Clinical Trial, 2016 | 8 |
| National Clinical Trial, 2017 | 7 |
| Pan African Clinical Trial Registry, 2016 | 7 |

*Reasons for Exclusion (Legend):*

1. Inadequate population (not evaluating single implant-supported crown surrounded by neighboring teeth in the maxillary aesthetic region);
2. Not evaluating PROMs measured by more than a 3-point scale or 3-item standardized questionnaire;
3. Not evaluating aesthetic outcomes;
4. Evaluating PROMs and ClinROs registered in different time points;
5. Inadequate study type;
6. Articles reported not in English;
7. Trial registered, not yet published;
8. Trial of published and included article.

**REFERENCES**

1. Abd el Aziz, N. F., Abd El-Rahman, A. R., El-Barbari, A. M., & Elarab, A. E. (2021). The esthetic effect of connective tissue graft addition around immediate dental implants in the esthetic zone: A randomized clinical trial. Journal of Osseointegration, 14(2), 97-106. <https://doi.org/10.23805/JO.2022.14.8>
2. Abduo, J., Gade, L., Gill, H., Judge, R., & Darby, I. (2017). A comparative study of encode protocol versus conventional protocol for restoring single implants: One-year prospective randomized controlled clinical trial. Clin Implant Dent Relat Res, 19(6), 1061-1067. <https://doi.org/10.1111/cid.12541>
3. Abduo, J., Lee, C. L., Sarfarazi, G., Xue, B., Judge, R., & Darby, I. (2021). Encode Protocol Versus Conventional Protocol for Single-Implant Restoration: A Prospective 2-Year Follow-Up Randomized Controlled Trial. J Oral Implantol, 47(1), 36-43. <https://doi.org/10.1563/aaid-joi-D-19-00150>
4. Abolhasani, M., Givehchian, P., Fathi, A., & Goudarzi, S. (2021). Relationship of Life Satisfaction and Satisfaction with Fixed Implant-Supported Prostheses in the Elderly [Original]. Journal of Iranian Dental Association, 33(1), 17-21. <https://doi.org/10.52547/jida.33.1.2.17>
5. Actrn. (2018a). Comparing the long-term clinical outcome of two designs of high strength ceramic (zirconia) crowns on dental implants [Trial registry record]. <http://www.who.int/trialsearch/Trial2.aspx?TrialID=ACTRN12618000614291>. <https://www.cochranelibrary.com/central/doi/10.1002/central/CN-02441222/full>
6. Actrn. (2018b). For replacing a single missing tooth with implant, is there a difference between the traditional and the new techniques for placing implants in relation to accuracy and restoration longevity [Trial registry record]. <http://www.who.int/trialsearch/Trial2.aspx?TrialID=ACTRN12618000622202>. <https://www.cochranelibrary.com/central/doi/10.1002/central/CN-02432778/full>
7. Actrn. (2018c). Impact of Alveolar Ridge Preservation on Implant Supported Restorations [Trial registry record]. <http://www.who.int/trialsearch/Trial2.aspx?TrialID=ACTRN12618000040268>. <https://www.cochranelibrary.com/central/doi/10.1002/central/CN-02432139/full>
8. Actrn. (2018d). Narrow diameter dental implant for replacing single missing back tooth [Trial registry record]. <https://trialsearch.who.int/Trial2.aspx?TrialID=ACTRN12618001016224>. <https://www.cochranelibrary.com/central/doi/10.1002/central/CN-01909464/full>
9. Actrn. (2019). Enamel Matrix Derivative (EMDOGAIN) for the treatment of gum recession and dental implant infection/ridge management [Trial registry record]. <https://trialsearch.who.int/Trial2.aspx?TrialID=ACTRN12619000062123>. <https://www.cochranelibrary.com/central/doi/10.1002/central/CN-01950225/full>
10. Afrashtehfar, K. I. (2021). Conventional free-hand, dynamic navigation and static guided implant surgery produce similar short-term patient-reported outcome measures and experiences. Evid Based Dent, 22(4), 143-145. <https://doi.org/10.1038/s41432-021-0216-9>
11. Afrashtehfar, K. I., Igarashi, K., & Bryant, S. R. (2021). Canadian Dental Patients with a Single-Unit Implant-Supported Restoration in the Aesthetic Region of the Mouth: Qualitative and Quantitative Patient-Reported Outcome Measures (PROMs). Data, 6(8), 90. <https://www.mdpi.com/2306-5729/6/8/90>
12. Al-Dosari, A., Al-Rowis, R., Moslem, F., Alshehri, F., & Ballo, A. M. (2016). Esthetic outcome for maxillary anterior single implants assessed by different dental specialists. J Adv Prosthodont, 8(5), 345-353. <https://doi.org/10.4047/jap.2016.8.5.345>
13. Alberga, J., Stellingsma, K., Meijer, H., Oostenbrink, A., Vissink, A., & Raghoebar, G. (2019). Dental Implant Placement in Cleft Patients: A Case-Control Study on Clinical and Aesthetic Outcomes. Journal of Oral and Maxillofacial Surgery, 77(9), e13. <https://doi.org/10.1016/j.joms.2019.06.030>
14. Alberga, J. M., Stellingsma, K., Meijer, H. J. A., Oostenbrink, H. A., Vissink, A., & Raghoebar, G. M. (2020). Dental implant placement in alveolar cleft patients: a retrospective comparative study on clinical and aesthetic outcomes. Int J Oral Maxillofac Surg, 49(7), 952-959. <https://doi.org/10.1016/j.ijom.2020.02.007>
15. Allen, P. F., Lee, S., & Brady, P. (2017). Clinical and subjective evaluation of implants in patients with hypodontia: a two-year observation study. Clin Oral Implants Res, 28(10), 1258-1262. <https://doi.org/10.1111/clr.12951>
16. Aloy-Prosper, A., Penarrocha-Oltra, D., Penarrocha-Diago, M., Camacho-Alonso, F., & Penarrocha-Diago, M. (2016). Peri-implant Hard and Soft Tissue Stability in Implants Placed Simultaneously Versus Delayed with Intraoral Block Bone Grafts in Horizontal Defects: A Retrospective Case Series Study. Int J Oral Maxillofac Implants, 31(1), 133-141. <https://doi.org/10.11607/jomi.4026>
17. Aloy-Prosper, A., Penarrocha-Oltra, D., Penarrocha-Diago, M., Hernandez-Alfaro, F., & Penarrocha-Diago, M. (2018). Peri-implant Tissues and Patient Satisfaction After Treatment of Vertically Augmented Atrophic Posterior Mandibles with Intraoral Onlay Block Bone Grafts: A Retrospective 3-Year Case Series Follow-up Study. Int J Oral Maxillofac Implants, 33(1), 137-144. <https://doi.org/10.11607/jomi.4490>
18. Altay, M. A., Sindel, A., Tezerisener, H. A., Yildirimyan, N., & Ozarslan, M. M. (2019). Esthetic evaluation of implant-supported single crowns: a comparison of objective and patient-reported outcomes. Int J Implant Dent, 5(1), 2. <https://doi.org/10.1186/s40729-018-0153-3>
19. Amer, O., Shemais, N., El-Sayed, K. F., Saleh, H. A., & Darhous, M. (2024). Does Injectable Platelet-Rich Fibrin Combined With Autogenous Demineralized Dentine Enhance Alveolar Ridge Preservation? A Randomized Controlled Trial [; Early Access]. Clinical oral implants research. <https://doi.org/10.1111/clr.14372>
20. Amorfini, L., Pesce, P., Migliorati, M., Drago, S., Storelli, S., Romeo, E., & Menini, M. (2023). Implant rehabilitation of the esthetic area: A five-year retrospective study comparing conventional and fully guided surgery. Clin Implant Dent Relat Res, 25(3), 438-446. <https://doi.org/10.1111/cid.13200>
21. Andersson, B., Bergenblock, S., Furst, B., & Jemt, T. (2013). Long-term function of single-implant restorations: a 17- to 19-year follow-up study on implant infraposition related to the shape of the face and patients' satisfaction. Clin Implant Dent Relat Res, 15(4), 471-480. <https://doi.org/10.1111/j.1708-8208.2011.00381.x>
22. Angkaew, C., Serichetaphongse, P., Krisdapong, S., Dart, M. M., & Pimkhaokham, A. (2017). Oral health-related quality of life and esthetic outcome in single anterior maxillary implants. Clin Oral Implants Res, 28(9), 1089-1096. <https://doi.org/10.1111/clr.12922>
23. Antonious, M., Couso-Queiruga, E., Barwacz, C., Gonzalez-Martin, O., & Avila-Ortiz, G. (2021). Evaluation of a Minimally Invasive Alveolar Ridge Reconstruction Approach in Postextraction Dehiscence Defects: A Case Series. Int J Periodontics Restorative Dent, 41(3), 335-345. <https://doi.org/10.11607/prd.4785>
24. Arora, H., & Ivanovski, S. (2018a). Evaluation of the influence of implant placement timing on the esthetic outcomes of single tooth implant treatment in the anterior maxilla: A retrospective study. J Esthet Restor Dent, 30(4), 338-345. <https://doi.org/10.1111/jerd.12385>
25. Arora, H., & Ivanovski, S. (2018b). Immediate and early implant placement in single-tooth gaps in the anterior maxilla: A prospective study on ridge dimensional, clinical, and aesthetic changes. Clin Oral Implants Res, 29(11), 1143-1154. <https://doi.org/10.1111/clr.13378>
26. Attia, S., Schaaf, H., El Khassawna, T., Malhan, D., Mausbach, K., Howaldt, H. P., & Streckbein, P. (2019). Oral Rehabilitation of Hypodontia Patients Using an Endosseous Dental Implant: Functional and Aesthetic Results. J Clin Med, 8(10). <https://doi.org/10.3390/jcm8101687>
27. Azaripour, A., Sagheb, K., Stock, L., Schiegnitz, E., Esposito, M., & Al Nawas, B. (2023). The Use of a Soft Tissue Substitute at Immediate Postextractive Implants to Reduce Tissue Shrinkage. One-Year Results from a Randomized Controlled Trial. International Journal of Periodontics & Restorative Dentistry, 43(4), 461-+. <https://doi.org/10.11607/prd.5620>
28. Baghel, A. S., Sahoo, P. K., Sharma, J., Almutairi, F. J., Adhyaru, H. G., Chhatbar, R., & Bajoria, A. A. (2023). Assessment of Biograft-HT with I-PRF Graft in Immediate Dental Implant Placement. Journal of Pharmacy and Bioallied Sciences, 15, S1168-S1170. <https://doi.org/10.4103/jpbs.jpbs_178_23>
29. Baer, R. A., Nolken, R., Colic, S., Heydecke, G., Mirzakhanian, C., Behneke, A., Behneke, N., Gottesman, E., Ottria, L., Pozzi, A., Fugl, A., & Zechner, W. (2022). Immediately provisionalized tapered conical connection implants for single-tooth restorations in the maxillary esthetic zone: a 5-year prospective single-cohort multicenter analysis. Clin Oral Investig, 26(4), 3593-3604. <https://doi.org/10.1007/s00784-021-04328-2>
30. Baer, R. A., Shanaman, R., Clark, P. K., & Medley, M. (2013). A 3-year multicenter study of marginal bone level and soft tissue health of a 1-piece implant. Implant Dent, 22(4), 366-373. <https://doi.org/10.1097/ID.0b013e31829a16ec>
31. Baldi, N., Buti, J., Mensi, M., Alfonsi, F., Cinquini, C., Tonelli, P., & Barone, A. (2020). Xenogeneic dermal matrix versus autologous connective tissue graft versus no graft at abutment connection for improving aesthetics: 6-month outcomes of a randomised controlled trial [Journal article]. Clinical trials in dentistry, 2(2), 49‐62. <https://doi.org/10.36130/CTD.03.2020.05>
32. Barausse, C., Esposito, M., Colombelli, F., Bellini, P., Buti, J., & Felice, P. (2020). Crestal or 1.5 mm subcrestal positioning of transmucosal dental implants with cemented or screw-retained crowns in posterior jaws: 4-month data from a single-centre randomised controlled trial [Journal article]. Clinical trials in dentistry, 2(2), 19‐33. <https://doi.org/10.36130/CTD.03.2020.03>
33. Barausse, C., Esposito, M., Pistilli, R., Buti, J., & Felice, P. (2019). Immediate loading of 3 mm-diameter implants as an alternative to horizontal bone augmentation for placing normal diameter implants: four-month post- loading results from a multicentre randomised controlled trial [Journal article]. Clinical trials in dentistry, 1(1), 51‐66. <https://www.cochranelibrary.com/central/doi/10.1002/central/CN-02345245/full>
34. Barone, A., Toti, P., Marconcini, S., Derchi, G., Saverio, M., & Covani, U. (2016). Esthetic Outcome of Implants Placed in Fresh Extraction Sockets by Clinicians with or without Experience: A Medium-Term Retrospective Evaluation. Int J Oral Maxillofac Implants, 31(6), 1397-1406. <https://doi.org/10.11607/jomi.4646>
35. Barwacz, C. A., Shah, K. C., Bittner, N., Parker, W., Neumeier, T. T., Thalji, G. N., & De Kok, I. J. (2021). A Retrospective, Multicenter, Cross-Sectional Case Series Study Evaluating Outcomes of CAD/CAM Abutments on Implants from Four Manufacturers: 4-Year Mean Follow-up. Int J Oral Maxillofac Implants, 36(5), 966-976. <https://doi.org/10.11607/jomi.8840>
36. Beekmans, D. G., Beekmans, B. R., & Cune, M. S. (2017). Pink and White Esthetics of a New Zirconia Implant: A 6-Month to 8-Year Follow-Up. Int J Periodontics Restorative Dent, 37(4), 511-518. <https://doi.org/10.11607/prd.2705>
37. Beekmans, D. G., Beekmans, R. M. N., & Cune, M. S. (2018). [The pink and white aesthetics of a new zirconia implant]. Ned Tijdschr Tandheelkd, 125(7-8), 389-395. <https://doi.org/10.5177/ntvt.2018.07/08.18134> (De roze en witte esthetiek van een nieuw zirkoniumdioxide implantaat.)
38. Bin, Z., Lihua, S., Junhua, Z., Tongbin, L., Yusan, L., Caiyun, C., & Jun, L. (2020). Short-term effect comparison of a modified socket shield technique and conventional flapless immediate implant and immediate restoration in maxillary aesthetic area [Journal article]. Chinese journal of tissue engineering research, 24(34), 5514‐5519. <https://doi.org/10.3969/j.issn.2095.4344.2344>
39. Boardman, N., Darby, I., & Chen, S. (2016). A retrospective evaluation of aesthetic outcomes for single-tooth implants in the anterior maxilla. Clin Oral Implants Res, 27(4), 443-451. <https://doi.org/10.1111/clr.12593>
40. Bonde, M. J., Stokholm, R., Schou, S., & Isidor, F. (2013). Patient satisfaction and aesthetic outcome of implant-supported single-tooth replacements performed by dental students: a retrospective evaluation 8 to 12 years after treatment. Eur J Oral Implantol, 6(4), 387-395. <https://www.ncbi.nlm.nih.gov/pubmed/24570983>
41. Bonino, F., Steffensen, B., Natto, Z., Hur, Y., Holtzman, L. P., & Weber, H. P. (2018). Prospective study of the impact of peri-implant soft tissue properties on patient-reported and clinically assessed outcomes. J Periodontol, 89(9), 1025-1032. <https://doi.org/10.1002/JPER.18-0031>
42. Boon, L., De Mars, G., Favril, C., Duyck, J., Quirynen, M., & Vandamme, K. (2020). Esthetic evaluation of single implant restorations, adjacent single implant restorations, and implant-supported fixed partial dentures: A 1-year prospective study. Clin Implant Dent Relat Res, 22(1), 128-137. <https://doi.org/10.1111/cid.12882>
43. Bose, M. W. H., Hildebrand, D., Beuer, F., Wesemann, C., Schwerdtner, P., Pieralli, S., & Spies, B. C. (2020). Clinical Outcomes of Root-Analogue Implants Restored with Single Crowns or Fixed Dental Prostheses: A Retrospective Case Series. J Clin Med, 9(8). <https://doi.org/10.3390/jcm9082346>
44. Branzén, M., Eliasson, A., Arnrup, K., & Bazargani, F. (2015). Implant-Supported Single Crowns Replacing Congenitally Missing Maxillary Lateral Incisors: A 5-Year Follow-Up. Clin Implant Dent Relat Res, 17(6), 1134-1140. <https://doi.org/10.1111/cid.12233>
45. Bressan, E., Grusovin, M. G., D'Avenia, F., Neumann, K., Sbricoli, L., Luongo, G., & Esposito, M. (2017). The influence of repeated abutment changes on peri-implant tissue stability: 3-year post-loading results from a multicentre randomised controlled trial. Eur J Oral Implantol, 10(4), 373-390.
46. Cacaci, C., Ackermann, K. L., Barth, T., Kistler, S., Stiller, M., & Schlee, M. (2019). A non-interventional multicenter study to document the implants success and survival rates in daily dental practices of the CONELOG screw-line implant. Clin Oral Investig, 23(6), 2609-2616. <https://doi.org/10.1007/s00784-018-2646-0>
47. Cairo, F., Barbato, L., Tonelli, P., Batalocco, G., Pagavino, G., & Nieri, M. (2017). Xenogeneic collagen matrix versus connective tissue graft for buccal soft tissue augmentation at implant site. A randomized, controlled clinical trial. J Clin Periodontol, 44(7), 769-776. <https://doi.org/10.1111/jcpe.12750>
48. Cappare, P., Ferrini, F., Ruscica, C., Pantaleo, G., Tete, G., & Gherlone, E. F. (2021). Digital versus Traditional Workflow for Immediate Loading in Single-Implant Restoration: A Randomized Clinical Trial [Journal article]. Biology, 10(12). <https://doi.org/10.3390/biology10121281>
49. Chang, M., & Wennström, J. L. (2013). Soft tissue topography and dimensions lateral to single implant-supported restorations. a cross-sectional study. Clin Oral Implants Res, 24(5), 556-562. <https://doi.org/10.1111/j.1600-0501.2012.02422.x>
50. Checchi, V., Felice, P., Zucchelli, G., Barausse, C., Piattelli, M., Pistilli, R., Grandi, G., & Esposito, M. (2017). Wide diameter immediate post-extractive implants vs delayed placement of normal-diameter implants in preserved sockets in the molar region: 1-year post-loading outcome of a randomised controlled trial. Eur J Oral Implantol, 10(3), 263-278.
51. Chen, Z., Zhang, S., Zhou, J., & Liang, H. (2022). Immediate versus Delayed Implantation for Single-Tooth Restoration of Maxillary Anterior Teeth: A Comparative Analysis on Efficacy. Comput Math Methods Med, 2022, 4490335. <https://doi.org/10.1155/2022/4490335>
52. Cosyn, J., & De Rouck, T. (2009). Aesthetic outcome of single-tooth implant restorations following early implant placement and guided bone regeneration: crown and soft tissue dimensions compared with contralateral teeth. Clin Oral Implants Res, 20(10), 1063-1069. <https://doi.org/10.1111/j.1600-0501.2009.01746.x>
53. Cosyn, J., Eghbali, A., De Bruyn, H., Dierens, M., & De Rouck, T. (2012). Single implant treatment in healing versus healed sites of the anterior maxilla: an aesthetic evaluation. Clin Implant Dent Relat Res, 14(4), 517-526. <https://doi.org/10.1111/j.1708-8208.2010.00300.x>
54. Cosyn, J., Eghbali, A., Hanselaer, L., De Rouck, T., Wyn, I., Sabzevar, M. M., Cleymaet, R., & De Bruyn, H. (2013). Four modalities of single implant treatment in the anterior maxilla: a clinical, radiographic, and aesthetic evaluation. Clin Implant Dent Relat Res, 15(4), 517-530. <https://doi.org/10.1111/j.1708-8208.2011.00417.x>
55. Covani, U., Marconcini, S., Galassini, G., Cornelini, R., Santini, S., & Barone, A. (2007). Connective tissue graft used as a biologic barrier to cover an immediate implant. J Periodontol, 78(8), 1644-1649. <https://doi.org/10.1902/jop.2007.060461>
56. Ctri. (2023). To compare outcomes in patient of intertrochanteric fractures undergoing weight bearing at different intervals after fixation [Trial registry record]. <https://trialsearch.who.int/Trial2.aspx?TrialID=CTRI/2023/09/057233>. <https://www.cochranelibrary.com/central/doi/10.1002/central/CN-02599247/full>
57. Ctri. (2024). A Comparison of The Biological And Clinical Results Of Different Temporary Crowns On Immediate Loaded Implant In The Maxillary Esthetic Zone [Trial registry record]. <https://trialsearch.who.int/Trial2.aspx?TrialID=CTRI/2024/05/066676>. <https://www.cochranelibrary.com/central/doi/10.1002/central/CN-02704266/full>
58. Ctri. (2018). A clinical trial to assess the effect of change in bone levels following implant placement immediately after removal of tooth with and without socket shield (leaving a tooth root section) [Trial registry record]. <https://trialsearch.who.int/Trial2.aspx?TrialID=CTRI/2018/06/014610>. <https://www.cochranelibrary.com/central/doi/10.1002/central/CN-01906274/full>
59. Ctri. (2019). Soft and Hard Tissue Changes Following Placement of Implants Immediately after Extraction of teeth [Trial registry record]. <https://trialsearch.who.int/Trial2.aspx?TrialID=CTRI/2019/06/019723>. <https://www.cochranelibrary.com/central/doi/10.1002/central/CN-02065720/full>
60. Ctri. (2021). To evaluate and compare immediate implant placement along with provisionalization with and without Concentrated Growth Factor-enriched bone graft [Trial registry record]. <https://trialsearch.who.int/Trial2.aspx?TrialID=CTRI/2021/01/030848>. <https://www.cochranelibrary.com/central/doi/10.1002/central/CN-02240974/full>
61. Ctri. (2021). To evaluate and compare immediate implant placement along with provisionalization with and without Concentrated Growth Factor-enriched bone graft [Trial registry record]. <https://trialsearch.who.int/Trial2.aspx?TrialID=CTRI/2021/01/030848>. <https://www.cochranelibrary.com/central/doi/10.1002/central/CN-02240974/full>
62. Ctri. (2022). Comparison of influence of microgrooved grip and spiral implant with microgrooved abutment on peri-implant hard and soft tissues in A clinical trial [Trial registry record]. <https://trialsearch.who.int/Trial2.aspx?TrialID=CTRI/2022/09/045655>. <https://www.cochranelibrary.com/central/doi/10.1002/central/CN-02473288/full>
63. D'Avenia, F., Bressan, E., Grusovin, M. G., Neumann, K., Sbricoli, L., Luongo, G., Piombino, P., Buti, J., & Esposito, M. (2020). The impact of repeated abutment changes on peri-implant tissue stability: five-year post-loading results from a multicentre randomised controlled trial [Journal article]. Clinical trials in dentistry, 2(1), 27‐46. <https://www.cochranelibrary.com/central/doi/10.1002/central/CN-02345227/full>
64. Dai, Y., Xu, J., Han, X. H., Cui, F. Z., Zhang, D. S., & Huang, S. Y. (2021). Clinical efficacy of mineralized collagen (MC) versus anorganic bovine bone (Bio-Oss) for immediate implant placement in esthetic area: a single-center retrospective study. Bmc Oral Health, 21(1), 390. <https://doi.org/10.1186/s12903-021-01752-4>
65. De Angelis, P., Rella, E., Manicone, P. F., Liguori, M. G., De Rosa, G., Cavalcanti, C., Galeazzi, N., & D'Addona, A. (2023). Xenogeneic collagen matrix versus connective tissue graft for soft tissue augmentation at immediately placed implants: a prospective clinical trial [Article]. International Journal of Oral and Maxillofacial Surgery, 52(10), 1097-1105. <https://doi.org/10.1016/j.ijom.2023.01.019>
66. De Angelis, N., Felice, P., Pellegrino, G., Camurati, A., Gambino, P., & Esposito, M. (2011). Guided bone regeneration with and without a bone substitute at single post-extractive implants: 1-year post-loading results from a pragmatic multicentre randomised controlled trial. Eur J Oral Implantol, 4(4), 313-325.
67. De Rouck, T., Collys, K., & Cosyn, J. (2008). Immediate single-tooth implants in the anterior maxilla: a 1-year case cohort study on hard and soft tissue response. J Clin Periodontol, 35(7), 649-657. <https://doi.org/10.1111/j.1600-051X.2008.01235.x>
68. Delize, V., Bouhy, A., Lambert, F., & Lamy, M. (2019). Intrasubject comparison of digital vs. conventional workflow for screw-retained single-implant crowns: prosthodontic and patient-centered outcomes [Journal article]. Clinical oral implants research, 30(9), 892‐902. <https://doi.org/10.1111/clr.13494>
69. Drks. (2014). Survival of 3D-planned and template-based implants inserted depending on the pre-implant hard and soft tissue management - a randomized controlled clinical trial [Trial registry record]. <https://trialsearch.who.int/Trial2.aspx?TrialID=DRKS00005978>. <https://www.cochranelibrary.com/central/doi/10.1002/central/CN-01815686/full>
70. Drks. (2015). Randomized controlled clinical study on the Long-term success of implants and the prosthetic supply for rehabilkitation of single-tooth gaps in postertior tooth regions depending on the material selection for hybridabutment crowns [Trial registry record]. <https://trialsearch.who.int/Trial2.aspx?TrialID=DRKS00009628>. <https://www.cochranelibrary.com/central/doi/10.1002/central/CN-01865088/full>
71. Drks. (2018). Immediate implant placement with alveolar ridge preservation or partial root retention: a randomized controlled clinical trial assessing biological long-term complications, three-dimensional ridge alterations and patient-reported outcome measures [Trial registry record]. <https://trialsearch.who.int/Trial2.aspx?TrialID=DRKS00014324>. <https://www.cochranelibrary.com/central/doi/10.1002/central/CN-01899146/full>
72. Drks. (2020a). Guided bone regeneration of peri-implant defects with particulate versus soft-type block bone substitutes. A randomized controlled, single blinded clinical trial [Trial registry record]. <https://trialsearch.who.int/Trial2.aspx?TrialID=DRKS00020222>. <https://www.cochranelibrary.com/central/doi/10.1002/central/CN-02168777/full>
73. Drks. (2020b). A MULTICENTER RANDOMIZED CONTROLLED STUDY COMPARING EARLY IMPLANT PLACEMENT TO ALVEOLAR RIDGE PRESERVATION FOR SINGLE TOOTH REPLACEMENT IN THE ANTERIOR AREA [Trial registry record]. <https://trialsearch.who.int/Trial2.aspx?TrialID=DRKS00018875>. <https://www.cochranelibrary.com/central/doi/10.1002/central/CN-02067374/full>
74. Drks. (2021). Immediate implant placement in molar extraction sockets using fully tapered tissue level implants: a randomized controlled post-market clinical study in Germany, Hong Kong and Switzerland [Trial registry record]. <https://trialsearch.who.int/Trial2.aspx?TrialID=DRKS00027236>. <https://www.cochranelibrary.com/central/doi/10.1002/central/CN-02377851/full>
75. Drks. (2022a). Additive versus subtractive manufactured interim implantsupported restorations in terms of clinical, aesthetic, patient-reported outcomes as well as cost- and timeefficiency: a randomized clinical trial “Additive vs subtractive interim” [Trial registry record]. <https://trialsearch.who.int/Trial2.aspx?TrialID=DRKS00029049>. <https://www.cochranelibrary.com/central/doi/10.1002/central/CN-02410280/full>
76. Drks. (2022b). Patients' perception of the immediate restoration of posterior single implants by an innovative fully digital one-abutment/one-time concept (SafetyCrown) compared with the standard procedure (submerged healing) - A randomized controlled clinical trial [Trial registry record]. <https://trialsearch.who.int/Trial2.aspx?TrialID=DRKS00027484>. <https://www.cochranelibrary.com/central/doi/10.1002/central/CN-02429426/full>
77. Drks. (2023). Esthetic Outcome, Clinical Performance, Patient Perception and Cost-Time Efficiency of Implant-supported Single Crowns influenced by the Prosthetic Workflow – a Randomized Clinical Trial in the Esthetic Zone. <https://trialsearch.who.int/Trial2.aspx?TrialID=DRKS00032124>.
78. Edinger, D. H., & Beuer, F. (2021). Rehabilitation of one-piece screw-retained implant crowns placed at second-stage surgery-a retrospective patient series. Clin Oral Investig, 25(3), 1345-1351. <https://doi.org/10.1007/s00784-020-03442-x>
79. Elaskary, A., Abdelrahman, H., Elfahl, B., Elsabagh, H., El-Kimary, G., & Ghallab, N. A. (2023). Immediate Implant Placement in Intact Fresh Extraction Sockets Using Vestibular Socket Therapy Versus Partial Extraction Therapy in the Esthetic Zone: A Randomized Clinical Trial [Article]. International Journal of Oral and Maxillofacial Implants, 38(3), 468-478. <https://doi.org/10.11607/JOMI.9973>
80. Ellithy, A. A., El-Tonsy, M. M., Ghouraba, S. F., El-Fahl, B. N., Elaskary, A., & Elfana, A. (2024). Immediate implant placement in compromised extraction sockets using vestibular socket therapy with acellular dermal matrix versus connective tissue grafts in the esthetic zone: a randomized controlled clinical trial [Journal article]. Clinical oral investigations, 28(12), 664. <https://doi.org/10.1007/s00784-024-06065-8>
81. Esposito, M., Barausse, C., Bonifazi, L., Piattelli, M., Pistilli, R., Ferri, A., & Felice, P. (2020). Wide-diameter immediate post-extraction implants versus socket preservation and delayed placement of normal-diameter implants in the molar region: 5-year postloading outcome of a randomised controlled trial [Journal article]. Clinical trials in dentistry, 2(4), 21‐37. <https://doi.org/10.36130/CTD.05.2020.03>
82. Esposito, M., Bressan, E., Grusovin, M. G., D'Avenia, F., Neumann, K., Sbricoli, L., & Luongo, G. (2017). Do repeated changes of abutments have any influence on the stability of peri-implant tissues? One-year post-loading results from a multicentre randomised controlled trial. Eur J Oral Implantol, 10(1), 57-72.
83. Esposito, M., Cardaropoli, D., Gobbato, L., Scutellà, F., Fabianelli, A., Mascellani, S., Delli Ficorelli, G., Mazzocco, F., Sbricoli, L., & Trullenque-Eriksson, A. (2018). The role of dental implant abutment design on the aesthetic outcome: preliminary 3-month post-loading results from a multicentre split-mouth randomised controlled trial comparing two different abutment designs [Journal article]. European journal of oral implantology, 11(1), 77‐87. <https://www.cochranelibrary.com/central/doi/10.1002/central/CN-01603718/full>
84. Esposito, M., Grufferty, B., Papavasiliou, G., Dominiak, M., Trullenque-Eriksson, A., & Heinemann, F. (2018). Immediate loading of occluding definitive partial fixed prostheses vs non-occluding provisional restorations - 3-year post-loading results from a pragmatic multicentre randomised controlled trial. Eur J Oral Implantol, 11(3), 309-320.
85. Esposito, M., Zucchelli, G., Cannizzaro, G., Checchi, L., Barausse, C., Trullenque-Eriksson, A., & Felice, P. (2017). Immediate, immediate-delayed (6 weeks) and delayed (4 months) post-extractive single implants: 1-year post-loading data from a randomised controlled trial. Eur J Oral Implantol, 10(1), 11-26.
86. Fabbri, G., Fradeani, M., Dellificorelli, G., De Lorenzi, M., Zarone, F., & Sorrentino, R. (2017). Clinical Evaluation of the Influence of Connection Type and Restoration Height on the Reliability of Zirconia Abutments: A Retrospective Study on 965 Abutments with a Mean 6-Year Follow-Up. Int J Periodontics Restorative Dent, 37(1), 19-31. <https://doi.org/10.11607/prd.2974>
87. Fava, J., Lin, M., Zahran, M., & Jokstad, A. (2015). Single implant-supported crowns in the aesthetic zone: patient satisfaction with aesthetic appearance compared with appraisals by laypeople and dentists. Clin Oral Implants Res, 26(10), 1113-1120. <https://doi.org/10.1111/clr.12412>
88. Felice, P., Barausse, C., Buti, J., Gessaroli, M., & Esposito, M. (2020). Immediate, early (6 weeks) and delayed (4 months) single post- extractive implants: 3-year post-loading data from a randomised controlled trial [Journal article]. Clinical trials in dentistry, 1(1), 5‐23. <https://www.cochranelibrary.com/central/doi/10.1002/central/CN-02345230/full>
89. Felice, P., Zucchelli, G., Cannizzaro, G., Barausse, C., Diazzi, M., Trullenque-Eriksson, A., & Esposito, M. (2016). Immediate, immediate-delayed (6 weeks) and delayed (4 months) post-extractive single implants: 4-month post-loading data from a randomised controlled trial. Eur J Oral Implantol, 9(3), 233-247.
90. Fenner, N., Hämmerle, C. H., Sailer, I., & Jung, R. E. (2016). Long-term clinical, technical, and esthetic outcomes of all-ceramic vs. titanium abutments on implant supporting single-tooth reconstructions after at least 5 years. Clin Oral Implants Res, 27(6), 716-723. <https://doi.org/10.1111/clr.12654>
91. Ferreira, F. N. H., Moreira Neto, J. J. S., de Negreiros, W. A., Kurita, L. M., & Silva, P. G. B. (2023). Evaluation of extra-narrow diameter implants in the oral rehabilitation of young patients after oral trauma: A prospective study. Dent Traumatol, 39(3), 248-256. <https://doi.org/10.1111/edt.12822>
92. Fonseca, M., Molinero-Mourelle, P., Forrer, F. A., Schnider, N., Hicklin, S. P., Schimmel, M., & Brägger, U. (2021). Clinical performance of implant crowns with customized zirconia abutments: A prospective cohort study with a 4.5- to 8.8-year follow-up. Clin Oral Implants Res, 32(7), 853-862. <https://doi.org/10.1111/clr.13761>
93. Foong, A. L. Y., Tey, V. H. S., Tan, K. B. C., Teoh, K. H., & Tan, K. (2022). Esthetic Evaluation of Anterior Implant-Supported Single Crowns: A Comparison Between Patients and Dentists. Int J Prosthodont, 35(4), 396-404. <https://doi.org/10.11607/ijp.8032>
94. Fu, L., Liu, G., Wu, X., Zhu, Z., Sun, H., & Xia, H. (2021). Patient-reported outcome measures of edentulous patients restored with single-implant mandibular overdentures: A systematic review. J Oral Rehabil, 48(1), 81-94. <https://doi.org/10.1111/joor.13103>
95. Fügl, A., Zechner, W., Pozzi, A., Heydecke, G., Mirzakhanian, C., Behneke, N., Behneke, A., Baer, R. A., Nölken, R., Gottesman, E., & Colic, S. (2017). An open prospective single cohort multicenter study evaluating the novel, tapered, conical connection implants supporting single crowns in the anterior and premolar maxilla: interim 1-year results. Clin Oral Investig, 21(6), 2133-2142. <https://doi.org/10.1007/s00784-016-2003-0>
96. Fürhauser, R., Fürhauser, L., Fürhauser, N., Pohl, V., Pommer, B., & Haas, R. (2022). Bucco-palatal implant position and its impact on soft tissue level in the maxillary esthetic zone. Clin Oral Implants Res, 33(11), 1125-1134. <https://doi.org/10.1111/clr.13995>
97. García-Minguillán, G., Del Río, J., Preciado, A., Lynch, C. D., & Castillo-Oyagüe, R. (2020). Impact of the retention system of implant fixed dental restorations on the peri-implant health, state of the prosthesis, and patients' oral health-related quality of life. J Dent, 94, 103298. <https://doi.org/10.1016/j.jdent.2020.103298>
98. Gjelvold, B., Chrcanovic, B. R., Bagewitz, I. C., Kisch, J., Albrektsson, T., & Wennerberg, A. (2017). Esthetic and Patient-Centered Outcomes of Single Implants: A Retrospective Study. Int J Oral Maxillofac Implants, 32(5), 1065-1073. <https://doi.org/10.11607/jomi.5495>
99. Gjelvold, B., Kisch, J., Mohammed, D. J. H., Chrcanovic, B. R., Albrektsson, T., & Wennerberg, A. (2020). Immediate Loading of Single Implants, Guided Surgery, and Intraoral Scanning: A Nonrandomized Study. Int J Prosthodont, 33(5), 513-522. <https://doi.org/10.11607/ijp.6701>
100. Groenendijk, E., Bronkhorst, E. M., & Meijer, G. J. (2021). Does the pre-operative buccal soft tissue level at teeth or gingival phenotype dictate the aesthetic outcome after flapless immediate implant placement and provisionalization? Analysis of a prospective clinical case series. Int J Implant Dent, 7(1), 84. <https://doi.org/10.1186/s40729-021-00366-3>
101. Hamdy, A., Ibrahim, S. S. A., Ghalwash, D., & Adel-Khattab, D. (2024). Volumetric assessment of volume stable collagen matrix in maxillary single implant site development: A randomized controlled clinical trial. Clin Implant Dent Relat Res, 26(5), 930-941. <https://doi.org/10.1111/cid.13353>
102. Han, X., Qi, C., Guo, P., Zhang, S., Xu, Y., Lv, G., Li, Y., & Li, C. (2021). Whole-Process Digitalization-Assisted Immediate Implant Placement and Immediate Restoration in the Aesthetic Zone: A Prospective Study. Med Sci Monit, 27, e931544. <https://doi.org/10.12659/msm.931544>
103. Hartlev, J., Kohberg, P., Ahlmann, S., Andersen, N. T., Schou, S., & Isidor, F. (2014). Patient satisfaction and esthetic outcome after immediate placement and provisionalization of single-tooth implants involving a definitive individual abutment. Clin Oral Implants Res, 25(11), 1245-1250. <https://doi.org/10.1111/clr.12260>
104. Hashemi, A. M., Hashemi, H. M., Siadat, H., Shamshiri, A., Afrashtehfar, K. I., & Alikhasi, M. (2022). Fully Digital versus Conventional Workflows for Fabricating Posterior Three-Unit Implant-Supported Reconstructions: a Prospective Crossover Clinical Trial [Journal article]. International journal of environmental research and public health, 19(18). <https://doi.org/10.3390/ijerph191811456>
105. Heinemann, F., Grufferty, B., Papavasiliou, G., Dominiak, M., García, J. J., Trullenque-Eriksson, A., & Esposito, M. (2016). Immediate occluding definitive partial fixed prosthesis versus non-occluding provisional restorations - 4-month post-loading results from a pragmatic multicenter randomised controlled trial. Eur J Oral Implantol, 9(1), 47-56.
106. Heydecke, G., Mirzakhanian, C., Behneke, A., Behneke, N., Fügl, A., Zechner, W., Baer, R. A., Nölken, R., Gottesman, E., Colic, S., Ottria, L., & Pozzi, A. (2019). A prospective multicenter evaluation of immediately functionalized tapered conical connection implants for single restorations in maxillary anterior and premolar sites: 3-year results. Clin Oral Investig, 23(4), 1877-1885. <https://doi.org/10.1007/s00784-018-2614-8>
107. Hof, M., Pommer, B., Ambros, H., Jesch, P., Vogl, S., & Zechner, W. (2015). Does Timing of Implant Placement Affect Implant Therapy Outcome in the Aesthetic Zone? A Clinical, Radiological, Aesthetic, and Patient-Based Evaluation. Clin Implant Dent Relat Res, 17(6), 1188-1199. <https://doi.org/10.1111/cid.12212>
108. Hof, M., Pommer, B., Strbac, G. D., Sütö, D., Watzek, G., & Zechner, W. (2013). Esthetic evaluation of single-tooth implants in the anterior maxilla following autologous bone augmentation. Clin Oral Implants Res, 24 Suppl A100, 88-93. <https://doi.org/10.1111/j.1600-0501.2011.02381.x>
109. Hof, M., Tepper, G., Koller, B., Krainhöfner, M., Watzek, G., & Pommer, B. (2014). Esthetic evaluation of single-tooth implants in the anterior mandible. Clin Oral Implants Res, 25(9), 1022-1026. <https://doi.org/10.1111/clr.12210>
110. Hof, M., Umar, N., Budas, N., Seemann, R., Pommer, B., & Zechner, W. (2018). Evaluation of implant esthetics using eight objective indices-Comparative analysis of reliability and validity. Clin Oral Implants Res, 29(7), 697-706. <https://doi.org/10.1111/clr.13261>
111. Hosseini, M., Worsaae, N., & Gotfredsen, K. (2022). A 5-year randomized controlled trial comparing zirconia-based versus metal-based implant-supported single-tooth restorations in the premolar region. Clin Oral Implants Res, 33(8), 792-803. <https://doi.org/10.1111/clr.13960>
112. Hosseini, M., Worsaae, N., Schiodt, M., & Gotfredsen, K. (2011). A 1-year randomised controlled trial comparing zirconia versus metal-ceramic implant supported single-tooth restorations. Eur J Oral Implantol, 4(4), 347-361.
113. Hosseini, M., Worsaae, N., Schiødt, M., & Gotfredsen, K. (2013). A 3-year prospective study of implant-supported, single-tooth restorations of all-ceramic and metal-ceramic materials in patients with tooth agenesis. Clin Oral Implants Res, 24(10), 1078-1087. <https://doi.org/10.1111/j.1600-0501.2012.02514.x>
114. Huber, S., Zeltner, M., Hämmerle, C. H. F., Jung, R. E., & Thoma, D. S. (2018). Non-interventional 1-year follow-up study of peri-implant soft tissues following previous soft tissue augmentation and crown insertion in single-tooth gaps. J Clin Periodontol, 45(4), 504-512. <https://doi.org/10.1111/jcpe.12865>
115. Hurtgen, A., Seidel, L., Manni, L. L., Liegeois, L., Lecloux, G., & Lambert, F. (2023). Clinical and radiographic assessment of circular versus triangular cross-section neck implants in the posterior maxilla: Five-year follow-up of a randomized controlled trial. Clin Oral Implants Res, 34(7), 698-706. <https://doi.org/10.1111/clr.14082>
116. Huynh-Ba, G., Hoders, A. B., Meister, D. J., Prihoda, T. J., Mills, M. P., Mealey, B. L., & Cochran, D. L. (2019). Esthetic, clinical, and radiographic outcomes of two surgical approaches for single implant in the esthetic area: 1-year results of a randomized controlled trial with parallel design. Clin Oral Implants Res, 30(8), 745-759. <https://doi.org/10.1111/clr.13458>
117. Huynh-Ba, G., Meister, D. J., Hoders, A. B., Mealey, B. L., Mills, M. P., Oates, T. W., Cochran, D. L., Prihoda, T. J., & McMahan, C. A. (2016). Esthetic, clinical and patient-centered outcomes of immediately placed implants (Type 1) and early placed implants (Type 2): preliminary 3-month results of an ongoing randomized controlled clinical trial. Clin Oral Implants Res, 27(2), 241-252. <https://doi.org/10.1111/clr.12577>
118. Isrctn. (2020a). Aesthetic evaluation of different materials for implant-supported teeth [Trial registry record]. <https://trialsearch.who.int/Trial2.aspx?TrialID=ISRCTN85107274>. <https://www.cochranelibrary.com/central/doi/10.1002/central/CN-02188471/full>
119. Isrctn. (2020b). Evaluation of gum and bone changes after immediate dental placement with two different surgical techniques [Trial registry record]. <https://trialsearch.who.int/Trial2.aspx?TrialID=ISRCTN81931981>. <https://www.cochranelibrary.com/central/doi/10.1002/central/CN-02188469/full>
120. Irct20240212060982N. (2024). Comparison of the ideal position of the implant in the aesthetic area [Trial registry record]. <https://trialsearch.who.int/Trial2.aspx?TrialID=IRCT20240212060982N1>. <https://www.cochranelibrary.com/central/doi/10.1002/central/CN-02743793/full>
121. Joda, T., Ferrari, M., Bragger, U., & Zitzmann, N. U. (2018). Patient Reported Outcome Measures (PROMs) of posterior single-implant crowns using digital workflows: a randomized controlled trial with a three-year follow-up [Journal article]. Clinical oral implants research, 29(9), 954‐961. <https://doi.org/10.1111/clr.13360>
122. Jonker, B. P., Wolvius, E. B., van der Tas, J. T., Tahmaseb, A., & Pijpe, J. (2020). Esthetics and Patient-Reported Outcomes of Implants Placed with Guided Bone Regeneration and Complete Native Bone: A Prospective Controlled Clinical Trial. Int J Oral Maxillofac Implants, 35(2), 406-414. <https://doi.org/10.11607/jomi.7751>
123. Josefsson, E., & Lindsten, R. (2019). Treatment of missing maxillary lateral incisors: a clinical and aesthetic evaluation. Eur J Orthod, 41(3), 273-278. <https://doi.org/10.1093/ejo/cjy061>
124. Kolinski, M. L., Cherry, J. E., McAllister, B. S., Parrish, K. D., Pumphrey, D. W., & Schroering, R. L. (2014). Evaluation of a variable-thread tapered implant in extraction sites with immediate temporization: a 3-year multicenter clinical study. J Periodontol, 85(3), 386-394. <https://doi.org/10.1902/jop.2013.120638>
125. Kotsailidi, E. A., Tatakis, D. N., Chen, Y. W., Caton, J. G., Ercoli, C., Barmak, A. B., & Tsigarida, A. (2022). Comparison of maxillary tuberosity and palatal donor sites for soft tissue augmentation at implant placement: A pilot controlled clinical study. Int J Oral Implantol (Berl), 15(4), 353-365.
126. Kourkouta, S., Dedi, K. D., Paquette, D. W., & Mol, A. (2009). Interproximal tissue dimensions in relation to adjacent implants in the anterior maxilla: clinical observations and patient aesthetic evaluation. Clin Oral Implants Res, 20(12), 1375-1385. <https://doi.org/10.1111/j.1600-0501.2009.01761.x>
127. Krawiec, M., Hadzik, J., Olchowy, C., Dominiak, M., & Kubasiewicz-Ross, P. (2021). Aesthetic Outcomes of Early Occlusal Loaded SLA Dental Implants with Hydroxyl Ion Modified Surface-A 12 Months Prospective Study. Materials (Basel), 14(21). <https://doi.org/10.3390/ma14216353>
128. Kuchler, U., Chappuis, V., Gruber, R., Lang, N. P., & Salvi, G. E. (2016). Immediate implant placement with simultaneous guided bone regeneration in the esthetic zone: 10-year clinical and radiographic outcomes. Clin Oral Implants Res, 27(2), 253-257. <https://doi.org/10.1111/clr.12586>
129. Kumar, M., Sah, R. P., Kumari, R., Rupam, K. R., Priya, P., & Jha, M. (2024). Aesthetic Outcome and Patient Perception of Immediate vs. Delayed Loading of Implant-Supported Single Crowns: A Randomized Controlled Trial. Journal of Pharmacy and Bioallied Sciences, 16, S446-S448. <https://doi.org/10.4103/jpbs.jpbs_704_23>
130. Kunavisarut, C., Buranajanyakul, L., Kitisubkanchana, J., & Pumpaluk, P. (2020). A Pilot Study of Small-Diameter One-Piece Ceramic Implants Placed in Anterior Regions: Clinical and Esthetic Outcomes at 1-Year Follow-up. Int J Oral Maxillofac Implants, 35(5), 965-973. <https://doi.org/10.11607/jomi.8308>
131. Kunavisarut, C., Santivitoonvong, A., Chaikantha, S., Pornprasertsuk-Damrongsri, S., & Joda, T. (2022). Patient-reported outcome measures comparing static computer-aided implant surgery and conventional implant surgery for single-tooth replacement: A randomized controlled trial. Clin Oral Implants Res, 33(3), 278-290. <https://doi.org/10.1111/clr.13886>
132. Lambert, F., Eldafrawy, M., Bekaert, S., & Mainjot, A. (2020). One-tooth one-time (1T1T), immediate loading of posterior single implants with the final crown: 2-year results of a case series. Int J Oral Implantol (Berl), 13(4), 369-383.
133. Landes, C. A., Bündgen, L., Laudemann, K., Ghanaati, S., & Sader, R. (2012). Patient satisfaction after prosthetic rehabilitation of bone-grafted alveolar clefts with nonsubmerged ITI Straumann dental implants loaded at three months. Cleft Palate Craniofac J, 49(5), 601-608. <https://doi.org/10.1597/10-156>
134. Levine, R. A., Dias, D. R., Wang, P., & Araújo, M. G. (2022). Effect of Connective Tissue Graft Following Immediate Implant Placement on Esthetic Outcomes at Maxillary Central Incisor Sites: A Long-Term Cohort Study. Int J Periodontics Restorative Dent, 42(5), e143-e151. <https://doi.org/10.11607/prd.5773>
135. Li, K., Liu, F., Liu, P., Wei, C., & Li, X. (2023). Clinical Effect and Aesthetic Evaluation of Minimally Invasive Implant Therapy [Journal article]. Emergency medicine international, 2023. <https://doi.org/10.1155/2023/9917311>
136. Li, W., Ruan, N., Tian, Y., Li, S., Boldbaatar, D., & Badral, B. (2024). Advantages of using touch-controlled, minimally invasive implantation technique on soft tissue in the aesthetic zone of maxillary anterior teeth [Article]. Medicine (United States), 103(50), e40051. <https://doi.org/10.1097/MD.0000000000040051>
137. Li Manni, L., Lecloux, G., Rompen, E., Aouini, W., Shapira, L., & Lambert, F. (2020). Clinical and radiographic assessment of circular versus triangular cross-section neck Implants in the posterior maxilla: A 1-year randomized controlled trial. Clin Oral Implants Res, 31(9), 814-824. <https://doi.org/10.1111/clr.13624>
138. Li, X., Wu, B., Cheng, X., Li, Y., Xie, X., & Deng, F. (2019). Esthetic Evaluation of Implant-Supported Single Crowns: The Implant Restoration Esthetic Index and Patient Perception. J Prosthodont, 28(1), e51-e58. <https://doi.org/10.1111/jopr.12659>
139. Liu, H., Liu, R., Wang, M., & Yang, J. (2019). Immediate implant placement combined with maxillary sinus floor elevation utilizing the transalveolar approach and nonsubmerged healing for failing teeth in the maxillary molar area: A randomized controlled trial clinical study with one-year follow-up. Clin Implant Dent Relat Res, 21(3), 462-472. <https://doi.org/10.1111/cid.12783>
140. Lorenz, J., Giulini, N., Hölscher, W., Schwiertz, A., Schwarz, F., & Sader, R. (2019). Prospective controlled clinical study investigating long-term clinical parameters, patient satisfaction, and microbial contamination of zirconia implants. Clin Implant Dent Relat Res, 21(2), 263-271. <https://doi.org/10.1111/cid.12720>
141. Luo, R., Zhu, Z., Huang, J., & Ye, Y. (2022). The Esthetic Outcome of Interproximal Papilla Between Implant-Restored Unilateral and Bilateral Maxillary Central Incisors: A Cross-Sectional Comparative Study. Int J Oral Maxillofac Implants, 37(5), 1063-1070. <https://doi.org/10.11607/jomi.9563>
142. Meijndert, C. M., Raghoebar, G. M., Santing, H. J., Vissink, A., & Meijer, H. J. A. (2020). Performance of bone-level implants with conical connections in the anterior maxilla: A 5-year prospective cohort study. Clin Oral Implants Res, 31(2), 173-180. <https://doi.org/10.1111/clr.13553>
143. Meijndert, C. M., Raghoebar, G. M., Vissink, A., & Meijer, H. J. (2022). Bone Level Tapered Implants in the Maxillary Esthetic Zone: A 1-Year Prospective Case Series in Healed Sites. Int J Oral Maxillofac Implants, 37(1), 120-127. <https://doi.org/10.11607/jomi.9163>
144. Merli, M., Moscatelli, M., Mariotti, G., Pagliaro, U., Raffaelli, E., & Nieri, M. (2018). Comparing membranes and bone substitutes in a one-stage procedure for horizontal bone augmentation. Three-year post-loading results of a double-blind randomised controlled trial. Eur J Oral Implantol, 11(4), 441-452.
145. Missinne, K., Duyck, J., Naert, I., Quirynen, M., Bertrand, S., & Vandamme, K. (2021). Oral Implant Restorations By Undergraduate Students: An Up To 5-Years Clinical Outcome. Int J Prosthodont, 34(4), 433–440. <https://doi.org/10.11607/ijp.7215>
146. Momberger, N., Mukaddam, K., Zitzmann, N. U., Bornstein, M. A., Filippi, A., & Kühl, S. (2022). Esthetic and functional outcomes of narrow-diameter implants compared in a cohort study to standard diameter implants in the anterior zone of the maxilla. Quintessence Int, 53(6), 502-509. <https://doi.org/10.3290/j.qi.b2887673>
147. Nct. (2019). One-abutment One-time for Immediate Restoration Procedure in the Esthetic Zone [Trial registry record]. <https://clinicaltrials.gov/show/NCT04139512>. <https://www.cochranelibrary.com/central/doi/10.1002/central/CN-01994378/full>
148. Nct. (2020). Single Crown Supported by Short Implant Versus Standard Implant in Conjunction With Maxillary Sinus Floor Augmentation [Trial registry record]. <https://clinicaltrials.gov/show/NCT04518020>. <https://www.cochranelibrary.com/central/doi/10.1002/central/CN-02162914/full>
149. Nct. (2021). Volumetric Assessment of Volume Stable Collagen Matrix in Soft Tissue Ridge Augmentation [Trial registry record]. <https://clinicaltrials.gov/show/NCT04873830>. <https://www.cochranelibrary.com/central/doi/10.1002/central/CN-02289479/full>
150. Nct. (2024a). Immediate vs Conventional Loading for Early Implant Placement [Trial registry record]. <https://clinicaltrials.gov/ct2/show/NCT06526351>. <https://www.cochranelibrary.com/central/doi/10.1002/central/CN-02741616/full>
151. Nct. (2024b). Insertion Protocol of Convergent Transmucosal Design Implants [Trial registry record]. <https://clinicaltrials.gov/ct2/show/NCT06254885>. <https://www.cochranelibrary.com/central/doi/10.1002/central/CN-02680184/full>
152. Nct. (2024c). Socket Reconstruction Technique and Immediate Implant Placement With Two Different Bone Substitute Materials [Trial registry record]. <https://clinicaltrials.gov/ct2/show/NCT06517030>. <https://www.cochranelibrary.com/central/doi/10.1002/central/CN-02738240/full>
153. Nct. (2024d). Success and Survival Rate of Tranmucosal Versus Bone Level Implants: randomized Clinical Trial [Trial registry record]. <https://clinicaltrials.gov/ct2/show/NCT06252324>. <https://www.cochranelibrary.com/central/doi/10.1002/central/CN-02680115/full>
154. Nct. (2024e). The Use of a Porcine Collagen Matrix for the Prevention of Buccal Bone Wall During Implant Placement in the Aesthetic Zone [Trial registry record]. <https://clinicaltrials.gov/ct2/show/NCT06622759>. <https://www.cochranelibrary.com/central/doi/10.1002/central/CN-02766718/full>
155. Nejatidanesh, F., Moradpoor, H., & Savabi, O. (2016). Clinical outcomes of zirconia-based implant- and tooth-supported single crowns. Clin Oral Investig, 20(1), 169-178. <https://doi.org/10.1007/s00784-015-1479-3>
156. Nielsen, H. B., Schou, S., Bruun, N. H., & Starch-Jensen, T. (2022). Professional and patient-reported outcomes of two surgical approaches for implant-supported single-crown restoration: 1-year results of a randomized controlled clinical trial. Clin Oral Implants Res, 33(2), 197-208. <https://doi.org/10.1111/clr.13883>
157. Ntr. (2005). Single tooth replacement with dental implants in the aesthetic zone A randomized clinical trial of different implant designs and different times of restoration [Trial registry record]. <https://trialsearch.who.int/Trial2.aspx?TrialID=NTR422>. <https://www.cochranelibrary.com/central/doi/10.1002/central/CN-01825970/full>
158. Ntr. (2016). The effect of membranes on small, bone augmentations at dental implant placement [Trial registry record]. <https://trialsearch.who.int/Trial2.aspx?TrialID=NTR6137>. <https://www.cochranelibrary.com/central/doi/10.1002/central/CN-01882703/full>
159. Ntr. (2017). Alveolar ridge preservation with a xenograft and a collagen matrix or a free connective tissue graft versus spontaneous healing: a 1-year prospective randomized clinical trial [Trial registry record]. <https://trialsearch.who.int/Trial2.aspx?TrialID=NTR6685>. <https://www.cochranelibrary.com/central/doi/10.1002/central/CN-01887411/full>
160. Pactr. (2016). Evaluation of marginal adaptation, esthetics and patients' satisfaction of lithium disilicate and nano hybrid resin ceramic crowns over zirconia impla [Trial registry record]. <https://trialsearch.who.int/Trial2.aspx?TrialID=PACTR201606001620344>. <https://www.cochranelibrary.com/central/doi/10.1002/central/CN-01828097/full>
161. Papi, P., Penna, D., Di Murro, B., & Pompa, G. (2021). Clinical and volumetric analysis of peri-implant soft tissue augmentation using an acellular dermal matrix: A prospective cohort study. J Periodontol, 92(6), 803-813. <https://doi.org/10.1002/jper.20-0219>
162. Pascoal, A. L. d. B., Paiva, K. R. G., de Araujo, L. N. M., Marinho, L. C. N., Gurgel, B. C. d. V., Dantas, W. R. M., Oliveira, A. G. R. d. C., & Calderon, P. d. S. (2024). One-Piece Versus Two-Piece Abutments for Single Crowns in the Esthetic Zone: A Clinical Trial [; Early Access]. Clinical oral implants research. <https://doi.org/10.1111/clr.14370>
163. Patil, R., Gresnigt, M. M. M., Mahesh, K., Dilbaghi, A., & Cune, M. S. (2017). Esthetic Evaluation of Anterior Single-Tooth Implants with Different Abutment Designs-Patients' Satisfaction Compared to Dentists' Observations. J Prosthodont, 26(5), 395-398. <https://doi.org/10.1111/jopr.12423>
164. pdj, R. B. R. (2023). Volumetric Analysis of a collagen graft versus a graft of patient's gum for the treatment of implant defects in esthetic region: controlled clinical trial [Trial registry record]. <https://trialsearch.who.int/Trial2.aspx?TrialID=RBR-54pdj93>. <https://www.cochranelibrary.com/central/doi/10.1002/central/CN-02592067/full>
165. Pellicer, L. E., Rubio, J. L. M., Casañas, E., & Villar, A. C. (2024). Immediate implant placement influenced by musical flow: a prospective randomized controlled clinical trial. BMC Oral Health, 24(1), 628. <https://doi.org/10.1186/s12903-024-04366-8>
166. Pollini, A., Morton, D., Arunyanak, S. P., Harris, B. T., & Lin, W. S. (2020). Evaluation of esthetic parameters related to a single implant restoration by laypeople and dentists. J Prosthet Dent, 124(1), 94-99. <https://doi.org/10.1016/j.prosdent.2019.08.017>
167. Raes, F., Cosyn, J., & De Bruyn, H. (2013). Clinical, aesthetic, and patient-related outcome of immediately loaded single implants in the anterior maxilla: a prospective study in extraction sockets, healed ridges, and grafted sites. Clin Implant Dent Relat Res, 15(6), 819-835. <https://doi.org/10.1111/j.1708-8208.2011.00438.x>
168. Raes, S., Eghbali, A., Chappuis, V., Raes, F., De Bruyn, H., & Cosyn, J. (2018). A long-term prospective cohort study on immediately restored single tooth implants inserted in extraction sockets and healed ridges: CBCT analyses, soft tissue alterations, aesthetic ratings, and patient-reported outcomes. Clin Implant Dent Relat Res, 20(4), 522-530. <https://doi.org/10.1111/cid.12613>
169. Ren, S. X., Hu, X. L., Li, J. H., Jiang, X., & Lin, Y. (2016). [A minimally invasive extraction technique using Benex Extraction System in flapless immediate implant placement in anterior teeth]. Shanghai Kou Qiang Yi Xue, 25(3), 334-339.
170. Roccuzzo, A., Imber, J. C., Lempert, J., Hosseini, M., & Jensen, S. S. (2022). Narrow diameter implants to replace congenital missing maxillary lateral incisors: A 1-year prospective, controlled, clinical study. Clin Oral Implants Res, 33(8), 844-857. <https://doi.org/10.1111/clr.13966>
171. Rupchandani, R. (2021). Do immediately placed implants have better outcomes when placed with a minimal split-thickness envelope flap? Evid Based Dent, 22(4), 126-127. <https://doi.org/10.1038/s41432-021-0215-x>
172. Rutkowski, R., Smeets, R., Neuhöffer, L., Stolzer, C., Strick, K., Gosau, M., Sehner, S., Volz, K. U., & Henningsen, A. (2022). Success and patient satisfaction of immediately loaded zirconia implants with fixed restorations one year after loading. Bmc Oral Health, 22(1), 198. <https://doi.org/10.1186/s12903-022-02231-0>
173. Sala, L., Zufía, J., Blasi, G., & Carrillo-de-Albornoz, A. (2023). Clinical evaluation and patient related outcomes of one- and two-piece zirconia implants at five years of loading: A case series study. J Esthet Restor Dent, 35(4), 577-585. <https://doi.org/10.1111/jerd.13002>
174. Santhanakrishnan, M., Ramesh, N., Kamaleeshwari, R., & Subramanian, V. (2021). Variations in Soft and Hard Tissues following Immediate Implant Placement versus Delayed Implant Placement following Socket Preservation in the Maxillary Esthetic Region: A Randomized Controlled Clinical Trial. Biomed Res Int, 2021, 5641185. <https://doi.org/10.1155/2021/5641185>
175. Santing, H. J., Raghoebar, G. M., Vissink, A., den Hartog, L., & Meijer, H. J. (2013). Performance of the Straumann Bone Level Implant system for anterior single-tooth replacements in augmented and nonaugmented sites: a prospective cohort study with 60 consecutive patients. Clin Oral Implants Res, 24(8), 941-948. <https://doi.org/10.1111/j.1600-0501.2012.02486.x>
176. Sauvin, G., Nurdin, N., Bischof, M., & Kiliaridis, S. (2022). Assessment and aesthetic impact of a long-term vertical discrepancy between the single anterior maxillary implant-supported crown and adjacent teeth: A retrospective cross-sectional study. Clin Exp Dent Res, 8(5), 1109-1116. <https://doi.org/10.1002/cre2.629>
177. Scheyer, E. T., Richardson, C., Mandelaris, G., Pickering, S., Nevins, M., Pope, B., Janakievski, J., Toback, G., & Heard, R. H. (2017). Retrospective Study to Determine Patient Satisfaction of Immediately Placed and Provisionalized Implants in the Esthetic Zone From a US Private-Practice Research Network. Compend Contin Educ Dent, 38(2), e9-e12.
178. Schnider, N., Forrer, F. A., Brägger, U., & Hicklin, S. P. (2018). Clinical Performance of One-Piece, Screw-Retained Implant Crowns Based on Hand-Veneered CAD/CAM Zirconia Abutments After a Mean Follow-up Period of 2.3 Years. Int J Oral Maxillofac Implants, 33(1), 188-196. <https://doi.org/10.11607/jomi.5929>
179. Schropp, L., & Isidor, F. (2008). Timing of implant placement relative to tooth extraction. J Oral Rehabil, 35 Suppl 1, 33-43. <https://doi.org/10.1111/j.1365-2842.2007.01827.x>
180. Shadid, R. M. (2022). Immediate implant placement with socket shield technique in the maxilla: a prospective case series evaluation at 1-year follow-up. Head Face Med, 18(1), 17. <https://doi.org/10.1186/s13005-022-00324-3>
181. Shahdad, S., Makdissi, J., & Gambôa, A. (2023). Relationship Between Facial Bone Dimensions, Orofacial Implant Position, and Esthetic Outcomes of Single-Tooth Implants. Int J Prosthodont, 36(6), 668-673. <https://doi.org/10.11607/ijp.8052>
182. Shrivastava, R., Ladda, R., Patadiya, M. M. M., Gautam, J., Gupta, S., Jadhav, M. S., & Makkad, R. S. (2024). Effect of Advanced Platelet-Rich Fibrin and Concentrated Growth Factor on Tissues Around Implants in the Maxillary Anterior Region. Journal of Pharmacy and Bioallied Sciences, 16, S2191-S2193. <https://doi.org/10.4103/jpbs.jpbs_137_24>
183. Sicilia-Felechosa, A., Pereira-Fernández, A., García-Lareu, J., Bernardo-González, J., Sicilia-Blanco, P., & Cuesta-Fernández, I. (2020). Flapless immediate implant placement and provisionalization in periodontal patients: A retrospective consecutive case-series study of single-tooth sites with dehiscence-type osseous defects. Clin Oral Implants Res, 31(3), 229-238. <https://doi.org/10.1111/clr.13559>
184. Sun, L., Yang, M. M., Zhao, J. M., Zhang, X., & Qu, Z. (2020). [Analysis of the hard and soft tissue following immediate and early implant placement in the anterior area of maxilla]. Zhonghua Kou Qiang Yi Xue Za Zhi, 55(11), 857-863. <https://doi.org/10.3760/cma.j.cn112144-20200610-00328>
185. Suphanantachat, S., Thovanich, K., & Nisapakultorn, K. (2012). The influence of peri-implant mucosal level on the satisfaction with anterior maxillary implants. Clin Oral Implants Res, 23(9), 1075-1081. <https://doi.org/10.1111/j.1600-0501.2011.02268.x>
186. Tartaglia, G. M., Sidoti, E., & Sforza, C. (2011). A 3-year follow-up study of all-ceramic single and multiple crowns performed in a private practice: a prospective case series. Clinics (Sao Paulo), 66(12), 2063-2070. <https://doi.org/10.1590/s1807-59322011001200011>
187. Tavelli, L., Majzoub, J., Kauffmann, F., Rodriguez, M. V., Mancini, L., Chan, H. L., Kripfgans, O. D., Giannobile, W. V., Wang, H. L., & Barootchi, S. (2023). Coronally advanced flap versus tunnel technique for the treatment of peri-implant soft tissue dehiscences with the connective tissue graft: a randomized, controlled clinical trial [Journal article]. Journal of clinical periodontology, 50(7), 980‐995. <https://doi.org/10.1111/jcpe.13806>
188. Tavelli, L., Zucchelli, G., Stefanini, M., Rasperini, G., Wang, H. L., & Barootchi, S. (2023). Vertical soft tissue augmentation to treat implant esthetic complications: A prospective clinical and volumetric case series. Clin Implant Dent Relat Res, 25(2), 204-214. <https://doi.org/10.1111/cid.13188>
189. Taylor, E. J., Yuan, J. C., Lee, D. J., Harlow, R., Afshari, F. S., Knoernschild, K. L., Campbell, S. D., & Sukotjo, C. (2014). Are predoctoral students able to provide single tooth implant restorations in the maxillary esthetic zone? J Dent Educ, 78(5), 779-788.
190. Tian, J. H., Di, P., Lin, Y., Zhang, Y., Wei, D. H., & Cui, H. Y. (2017). [A pilot clinical study of immediate provisionalization with a chairside computer aided design and computer aided manufacture monolithic crown for single tooth immediate implant placement]. Zhonghua Kou Qiang Yi Xue Za Zhi, 52(1), 3-9. <https://doi.org/10.3760/cma.j.issn.1002-0098.2017.01.002>
191. Topçu, A. O., Yamalik, N., Güncü, G. N., Tözüm, T. F., El, H., Uysal, S., & Hersek, N. (2017). Implant-Site Related and Patient-Based Factors With the Potential to Impact Patients' Satisfaction, Quality of Life Measures and Perceptions Toward Dental Implant Treatment. Implant Dent, 26(4), 581-591. <https://doi.org/10.1097/id.0000000000000623>
192. Trimpou, G., Schwarz, F., Begić, A., Hess, P., Lermen, J., Keim, N., Obreja, K., & Parvini, P. (2022). Clinical performance of immediately placed and restored progressive-type implants in the esthetic zone: a prospective observational study. Int J Implant Dent, 8(1), 57. <https://doi.org/10.1186/s40729-022-00462-y>
193. Tymstra, N., Meijer, H. J., Stellingsma, K., Raghoebar, G. M., & Vissink, A. (2010). Treatment outcome and patient satisfaction with two adjacent implant-supported restorations in the esthetic zone. Int J Periodontics Restorative Dent, 30(3), 307-316.
194. Urban, I. A., Wessing, B., Alández, N., Meloni, S., González-Martin, O., Polizzi, G., Sanz-Sanchez, I., Montero, E., & Zechner, W. (2019). A multicenter randomized controlled trial using a novel collagen membrane for guided bone regeneration at dehisced single implant sites: Outcome at prosthetic delivery and at 1-year follow-up. Clin Oral Implants Res, 30(6), 487-497. <https://doi.org/10.1111/clr.13426>
195. Van Nimwegen, W. G., Goené, R. J., Van Daelen, A. C., Stellingsma, K., Raghoebar, G. M., & Meijer, H. J. (2016). Immediate implant placement and provisionalisation in the aesthetic zone. J Oral Rehabil, 43(10), 745-752. <https://doi.org/10.1111/joor.12420>
196. Vanlıoğlu, B. A., Kahramanoğlu, E., Yıldız, C., Ozkan, Y., & Kulak-Özkan, Y. (2014). Esthetic outcome evaluation of maxillary anterior single-tooth bone-level implants with metal or ceramic abutments and ceramic crowns. Int J Oral Maxillofac Implants, 29(5), 1130-1136. <https://doi.org/10.11607/jomi.3439>
197. Vilhjálmsson, V. H., Klock, K. S., Størksen, K., & Bårdsen, A. (2011). Aesthetics of implant-supported single anterior maxillary crowns evaluated by objective indices and participants' perceptions. Clin Oral Implants Res, 22(12), 1399-1403. <https://doi.org/10.1111/j.1600-0501.2010.02128.x>
198. Wang, X. C., Wang, Y., Xu, X. B., & Hao, X. H. (2022). [Evaluation of the effect of digital crown extension guide in aesthetic restoration of anterior teeth]. Shanghai Kou Qiang Yi Xue, 31(3), 260-264.
199. Wang, Y. M., Liu, X., & He, J. C. (2021). Clinical application of integrated angulated screw channel abutment crown in implant-supported rehabilitation of aesthetic area. Hua Xi Kou Qiang Yi Xue Za Zhi, 39(6), 712-717. <https://doi.org/10.7518/hxkq.2021.06.014>
200. Wiesner, G., Esposito, M., Worthington, H., & Schlee, M. (2010). Connective tissue grafts for thickening peri-implant tissues at implant placement. One-year results from an explanatory split-mouth randomised controlled clinical trial. Eur J Oral Implantol, 3(1), 27-35.
201. Wong, J. L., Tan, K. B. C., Teoh, K. H., & Sim, C. P. C. (2020). Spectrophotometric and Visual Evaluation of Maxillary Anterior Implant Crowns and Peri-implant Soft Tissues: A Retrospective Study. Int J Prosthodont, 33(3), 277-284. <https://doi.org/10.11607/ijp.6526>
202. Wu, S. Q., Lu, H. B., Wen, Z. X., Zhang, X. Y., & Chen, G. D. (2020). [Analysis of clinical aesthetic effect of buccal alveolar ridge preservation and connective tissue transplantation with single implant]. Shanghai Kou Qiang Yi Xue, 29(6), 617-622.
203. Xu, Y. M., Huang, H., Wang, L., Wu, Q. Q., Fu, G., & Li, J. (2019). [Comparison of clinical effects of a modified socket shield technique and the conventional immediate implant placement]. Hua Xi Kou Qiang Yi Xue Za Zhi, 37(5), 490-495. <https://doi.org/10.7518/hxkq.2019.05.007>
204. Yan, S. J., Zhou, C., Liu, J., Xu, X. N., Yang, Y., Chen, X., & Lan, J. (2019). [Clinical evaluation of the socket-shield technique for immediate implantation in the maxillary anterior region]. Hua Xi Kou Qiang Yi Xue Za Zhi, 37(6), 615-620. <https://doi.org/10.7518/hxkq.2019.06.008>
205. Zhang, S., Lu, X., & Chen, Z. (2022). Clinical Effects of Simultaneous Implant Placement in Hydraulic Maxillary Sinus Lift Without Bone Grafting. Altern Ther Health Med, 28(7), 111-119.
206. Zhu, J., Sun, W., Li, L., Li, H., Zou, Y., Huang, B., Ji, W., & Shi, B. (2023). Accuracy and patient-centered results of marker-based and marker-free registrations for dynamic computer-assisted implant surgery: A randomized controlled trial. Clinical oral implants research. <https://doi.org/10.1111/clr.14201>
207. Zucchelli, G., Felice, P., Mazzotti, C., Marzadori, M., Mounssif, I., Monaco, C., & Stefanini, M. (2018). 5-year outcomes after coverage of soft tissue dehiscence around single implants: A prospective cohort study. Eur J Oral Implantol, 11(2), 215-224.
208. Zucchelli, G., Mazzotti, C., Mounssif, I., Mele, M., Stefanini, M., & Montebugnoli, L. (2013). A novel surgical-prosthetic approach for soft tissue dehiscence coverage around single implant. Clin Oral Implants Res, 24(9), 957-962. <https://doi.org/10.1111/clr.12003>
209. Zuercher, A. N., Ioannidis, A., Hüsler, J., Mehl, A., Hämmerle, C. H. F., & Thoma, D. S. (2023). Randomized controlled pilot study assessing efficacy, efficiency, and patient-reported outcomes measures of chairside and labside single-tooth restorations. J Esthet Restor Dent, 35(1), 74-83. <https://doi.org/10.1111/jerd.12909>
210. Zuercher, A. N., Mancini, L., Naenni, N., Thoma, D. S., Strauss, F. J., & Jung, R. E. (2023). The L-shape technique in guided bone regeneration with simultaneous implant placement in the esthetic zone: A step-by-step protocol and a 2-14 year retrospective study. J Esthet Restor Dent, 35(1), 197-205. <https://doi.org/10.1111/jerd.12965>
211. Zuiderveld, E. G., Meijer, H. J., Vissink, A., & Raghoebar, G. M. (2019). Outcome of Treatment with Single Implants in Preserved Versus Nonpreserved Alveolar Ridges: A 1-year Cohort Study. Int J Oral Maxillofac Implants, 34(6), 1457-1465. <https://doi.org/10.11607/jomi.7367>
